# Curriculum-Based Deep Learning for Automated Segmental Left Ventricular Hypertrophy Classification Using Short-Axis Echocardiography

**DOI:** 10.3390/diagnostics16193157

**Published:** 2026-09-28

**Authors:** Rajeswari Periyasamy, Srinivasan Selvaraj, Francis Antony Selvi Pitchaimuthu, Aneesh Euprazia Lucas

**Affiliations:** 1 Department of Computer Science and Engineering, R.M.D Engineering College, RSM Nagar, Kavaraipettai 601206, India; 2 Department of Electrical and Electronics Engineering, R.V.S. College of Engineering, Dindigul 624005, India; 3 Department of Artificial Intelligence and Data Science, Vel Tech Rangarajan Dr. Sagunthala R&D Institute of Science and Technology, Chennai 600062, India

**Keywords:** left ventricular hypertrophy, echocardiography, myocardial segmentation, curriculum learning, deep learning, ConvNeXt-V2

## Abstract

**Background:** Accurate detection and classification of segmental left ventricular hypertrophy (LVH) are challenging due to rib and reverberation artifacts in two-dimensional short-axis (2D-SAX) echocardiography view. These artifacts lead to ambiguity in the boundaries of the myocardial region. Existing artificial intelligence techniques focus on global LVH detection compared to segmental LVH classification. **Methodology:** To solve the abovementioned problem, a curriculum-based left ventricular hypertrophy deep learning (LVH-DL) framework is proposed for segmental LVH classification. The proposed framework was tested with 2000 images of segmental LVH, which consists of (a) apical-423, (b) basal-382, (c) mid-ventricular-349, (d) diffuse-453, and (e) normal-393. The curriculum-based deep learning model consists of proposed algorithms such as (i) preprocessing of Mountaineering-Team-Based Optimization Deep Denoised Convolutional Neural Network (MTBO-DnCNN) for removing artifacts and enhancing the boundaries, (ii) segmentation of modified active contour segmentation (MACS) algorithm accurately extracting the myocardium region from the background, and (iii) classification of curriculum-based ConvNeXt-V2 model classifying segmental LVH using inter-channel features such as valvular structures and chamber size from the segmented myocardial region. **Results:** The proposed LVH-DL framework achieves a classification accuracy of about (a) 96.5% for apical, (b) 95% for basal, (c) 95.8% for mid-ventricular, (d) 97.2% for diffuse, and (e) 95% for normal. **Conclusions:** Thus, Thus, the proposed LVH-DL framework acts as a decision support tool for early identification of segmental LVH.

## 1. Introduction

Left ventricular hypertrophy (LVH) occurs due to wall thickness in the left ventricle of the human heart. The left ventricular wall thickness increases the myocardial stiffness. LVH causes irregular heart rhythms, arrhythmias, and heart attacks [1]. LVH is classified based on geometric wall patterns as two-tiered and four-tiered LVH. The two-tiered LVH is further classified as concentric hypertrophy (CH) and eccentric hypertrophy (EH). CH and EH arise due to an increase in pressure or volume leading to LV remodeling with abnormal sarcomere arrangement. CH arises due to increasing wall thickness, which leads to high pressure. EH arises due to increases in wall thickness and high diastolic volume [2]. Pressure overload leads to chronic high systolic wall stress; sarcomeres added in parallel (side-by-side) cells get wider; wall thickening is known as concentric hypertrophy. Volume overload leads to chronic high diastolic stretch; sarcomeres added in series (end-to-end) cells get longer; chamber dilation is known as eccentric hypertrophy.

According to the Dallas Heart Study, the four-tiered LVH is further classified based on different wall thickness patterns and chamber dimensions and they are thick hypertrophy (TH), dilated hypertrophy (DH), both thick and dilated hypertrophy (TDH) and indeterminate hypertrophy (IH) [3].

TH arises due to the disproportionate relationship between left ventricular hypertrophy (LVH) wall thickness and end-diastolic volume (EDV). DH results from the disproportionate relationship between EDV and the enlargement of LVH wall thickness. TDH is caused by increased wall thickness and EDV. IH develops due to an increase in LVH wall thickness or an enlarged EDV. LVH is classified based on various pathophysiological conditions, including genetic mutations, diastolic dysfunction, outflow tract obstruction, myocardial fibrosis, pressure overload, and protein deposition [4,5]. Additionally, LVH is categorized by segments of the left ventricle, specifically (i) basal septal hypertrophy (BSH), (ii) apical hypertrophy (AH), (iii) mid-ventricular hypertrophy (MH), and (iv) diffuse hypertrophy (DH) [6,7].

Basal septal hypertrophy (BSH) is characterized by thickening of the left ventricular wall in the upper interventricular septum. Apical hypertrophy (AH) occurs due to thickening of the left ventricular wall in the lower myocardial segments of the left ventricle. Mid-ventricular hypertrophy arises in the mid-ventricular myocardial segments of the left ventricle. Diffuse hypertrophy affects the entire thickness of the left ventricular myocardial wall segments. LVH is measured using 2D echocardiography images, electrocardiograms (ECGs), and cardiac magnetic resonance imaging. Electrocardiographic (ECG) assessment of LVH is inaccurate because the recorded voltage and depolarization vectors are influenced by factors such as electrode position, pericardial fluid, body weight, and body habitus [8]. In MRI, LVH is assessed by measuring the left ventricular mass. Experienced radiologists are required for accurate classification of LVH in MRI images; otherwise, there is a risk of misdiagnosis.

Echocardiography is a non-invasive technique for segmental LVH assessment that provides high diagnostic accuracy by visualizing left ventricular wall thicknesses in different segments. However, echocardiography has limitations such as (i) geometric variations in left ventricular shape, (ii) variability in left ventricular mass (LVM) due to the inexperience of the radiologist, (iii) poor visualization of LVH at the endocardial and epicardial borders, and (iv) limited detection of regional abnormalities in heterogeneous hypertrophy, which reduces diagnostic accuracy [9,10].

Left ventricular hypertrophy (LVH) is assessed using two-dimensional echocardiography, electrocardiography (ECG), and cardiac magnetic resonance (CMR). ECG-based methods are used for detecting LVH and provide prognostic information of cardiovascular risk; however, their sensitivity for anatomical LVH is limited [10]. Recent studies have also applied deep learning methods to chest radiographs to detect LVH-related structural abnormalities. A deep learning model was proposed for identifying severe LVH and dilated left-ventricular structural abnormalities from CXR images [11]. Thus, artificial intelligence is used for screening, but it does not provide the detailed regional visualization required for segmental LVH classification.

Deep learning models are applied [12] to classify severe LVH and dilated LVH from chest X-ray images. The ResNet32 deep learning architecture model is used to distinguish between hypertrophic cardiomyopathy (HCM) and hypertensive heart disease (HHD) from MRI images, utilizing T1 maps with the myocardial ring bounding box [13]. A 2D-CNN model is used for LVH detection from ECG signals [14]. Additionally, a geometric-calculating nomogram device is used for detecting LVH in hypertension through a multiple linear regression model. These methods diagnose LVH based on the entire left ventricular wall, while segmental LVH classification remains challenging due to inaccuracies in myocardial structure measurements from ECG and cardiac magnetic resonance imaging images. Therefore, the echocardiogram’s short-axis (SAX) view is used for segmental LVH classification based on left ventricular segments.

Electrocardiography (ECG), cardiac magnetic resonance (CMR), and echocardiography are established modalities for evaluating LVH and hypertrophic cardiomyopathy. ECG has low spatial resolution, and detection of LVH at an earlier stage is challenging. Cardiac-magnetic-resonance-imaging-based LVH detection is difficult due to unclear boundary regions of LVH, which leads to inaccurate delineation of subtle myocardial abnormalities or assessment of dynamic intracavitary gradients and LVH wall region.

Table 1 provides the various imaging modalities, techniques, and research gaps in LVH classification.

### 1.1. Motivations

Existing artificial-intelligence-based techniques have several limitations, such as semi-automated segmentation leading to inconsistencies in LV endocardial delineation and LV wall thickness measurements. Although deep learning models classify global left ventricular hypertrophy, they are not suitable for segmental LVH classification, resulting in inaccurate diagnoses. Deep learning methods for cardiac magnetic resonance imaging require large, annotated datasets and are computationally expensive. ECG-based deep learning models suffer from low sensitivity and indirect signatures. Hence, echocardiography-based images are used for segmental LVH classification.

In echocardiography, the heart is viewed in different axes, including the SAX (short-axis) view, PLAX (parasternal long-axis) view, A4C (apical 4-chamber) view, and A2C (apical 2-chamber) view. The SAX view slices the heart transversely, showing the mitral valve, papillary muscles, or apex. The PLAX view displays the left atrium, left ventricle, mitral valve, aortic valve, and aortic root, allowing for evaluation of chamber size, wall thickness, and valve motion. The A4C view displays all four chambers: the left and right atria and ventricles. The A2C view highlights the anterior and inferior walls of the left ventricle.

The SAX view provides cross-sectional images of the left and right ventricles at multiple levels, such as the basal septum, mid-cavity, and apical regions. The clear delineation of the endocardial and epicardial borders enables accurate assessment of regional myocardial wall thickness and localization of hypertrophy within specific myocardial segments for segmental left ventricular hypertrophy (LVH) classification. The A4C view reduces diagnostic accuracy in cases of basal septal hypertrophy due to physiological stress and incomplete visualization of left ventricular wall segments [25]. The A2C view may not fully capture the entire intraventricular septum [26]. The PLAX view provides global measurements of the left ventricle but has limited access to the apical region. Due to its imaging plane, PLAX view reduced sensitivity for detecting apical or localized patterns of hypertrophy [27,28]. Therefore, the SAX view is preferred for segmental LVH classification.

**Figure 1:**
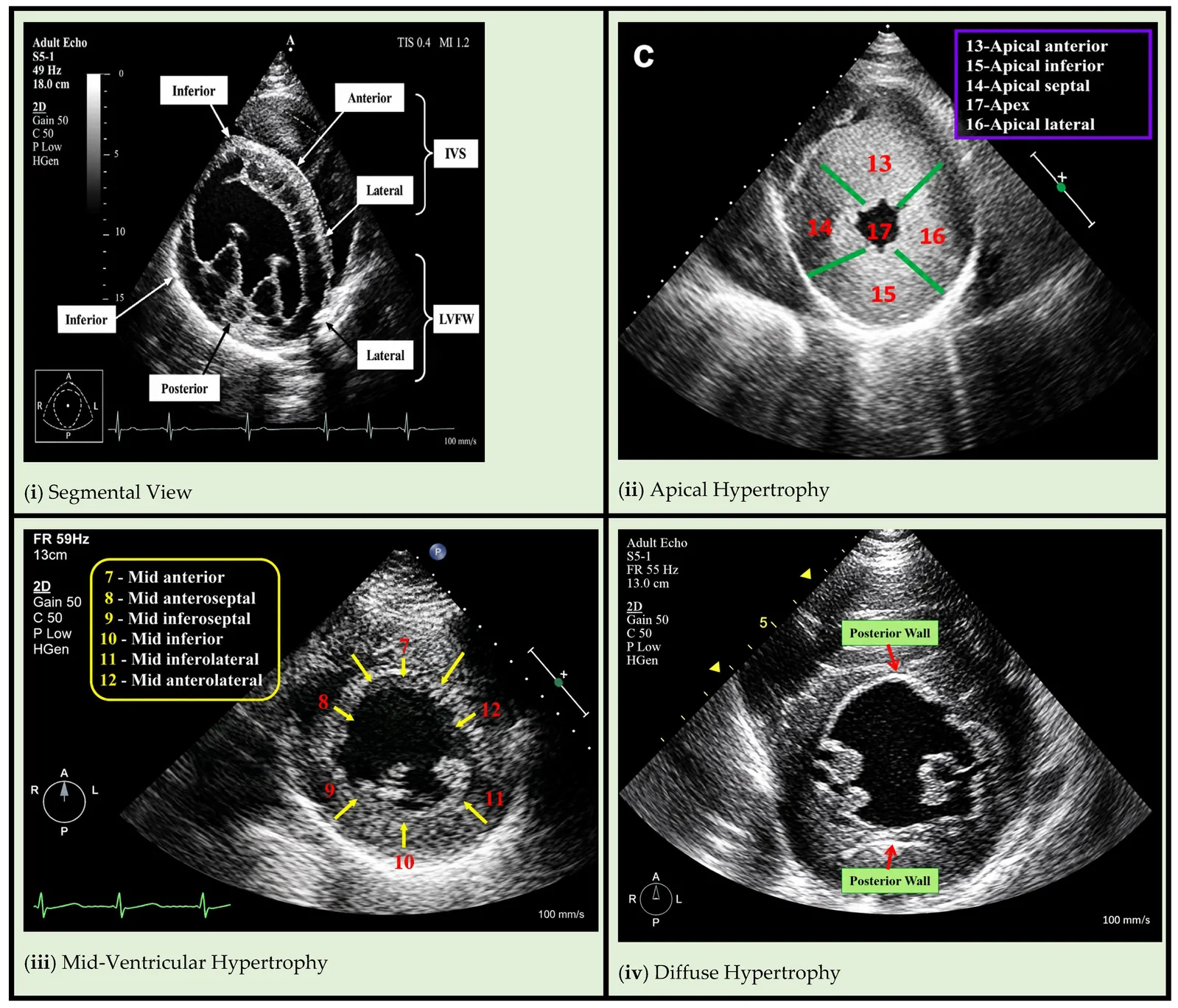

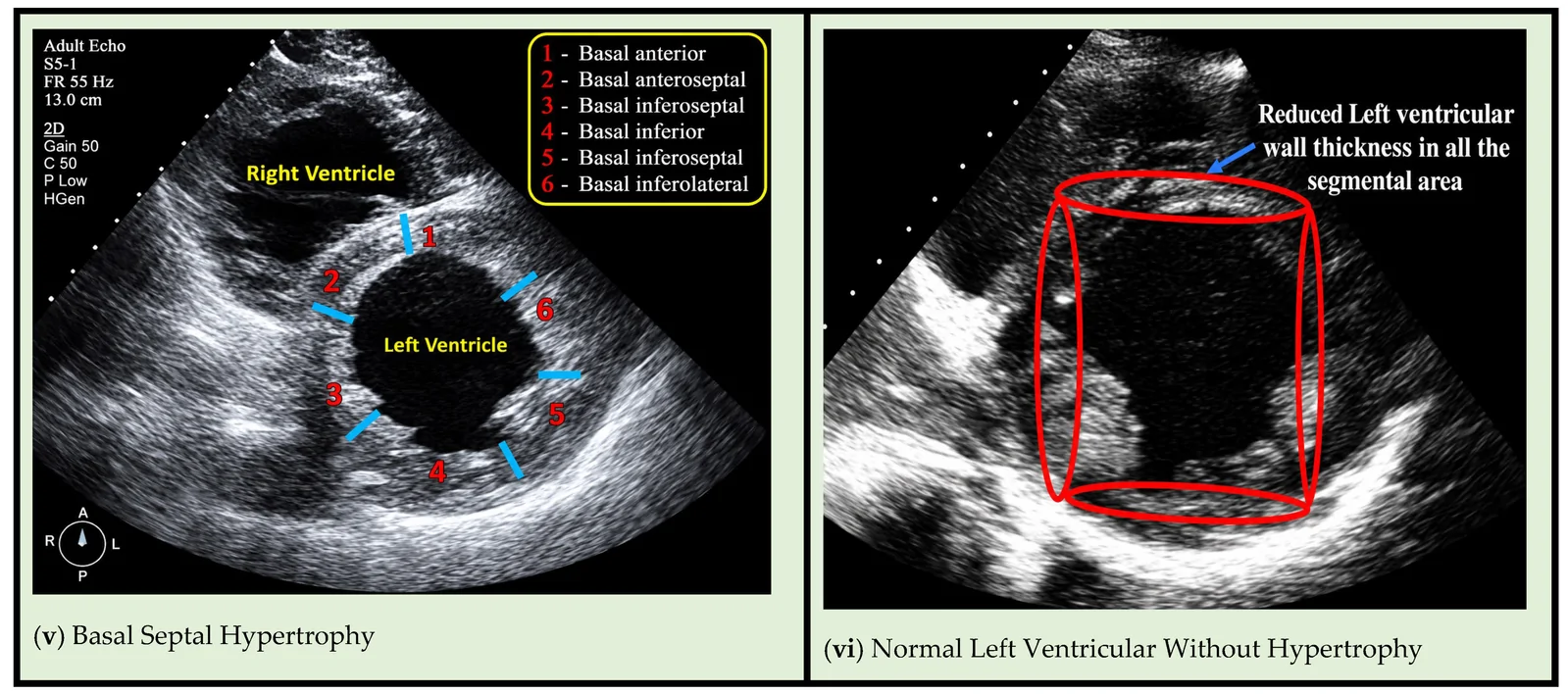
Segments and types of left ventricular hypertrophy based on left ventricular segments in 2D echocardiographic SAX view [30]. LV: Left ventricular, RV: right ventricular, AS: anterior septum, IS: inferior septum, and IVS: intraventricular septum.

Figure 1 shows the segmental short-axis view of a 2D echocardiogram, and types of LVH with damage on the left ventricular wall in different segments. LVH is diagnosed through an electrocardiogram (ECG), cardiac magnetic resonance imaging, and echocardiogram with less accuracy [29].

### 1.2. Proposed Contributions

These gaps can be addressed using the LVH-DL integrated framework which consists of Denoised Convolutional Neural Network (MTBO-DnCNN) for artifact suppression, a modified active contour (MAC) algorithm for precise segmentation, and curriculum-based deep learning models for robust segmental LVH classification in 2D echocardiographic SAX images.

1. To enhance the left ventricular wall region and reduce the reverberation and rib artifacts in 2D echocardiographic images the Mountaineering-Team-Based Optimization Denoised Convolutional Neural Network (MTBO-DnCNN) algorithm is proposed which detects the hypertrophy regions based on perspective projection of the wall regions. Perspective projection refers to a visualization of a region in 3D model. The human eye perceives a 3D structured wall surface from a 2D surface region.
2. To segment the left ventricular myocardial region more accurately from the SAX 2D echocardiographic image, modified active contour segmentation (MACS) algorithm is proposed, which segments the wall region and measures the thickness of the left ventricular wall in the apical, basal, and mid-ventricular regions of the LV segments effectively due to the spline focus on minimization energy. Splines represent the boundaries of the wall region and energy minimization adjusts splines for accurate boundary extraction through energy function.
3. The proposed framework performs five-class segmental LVH classification such as (i) basal septal hypertrophy, (ii) apical hypertrophy, (iii) mid-ventricular hypertrophy, (iv) diffuse hypertrophy, and (v) normal myocardium. To learn heterogeneous LVH patterns, a curriculum-based deep learning (CDL) framework is developed. The framework evaluates three curriculum-learning strategies such as Self-Paced Curriculum Learning (SP-CL), Progressive Learning–Curriculum Learning (PL-CL), and Teacher–Student Curriculum Learning (TS-CL).

In this paper, Section 2 describes the materials and methods. Section 3 presents the results and discussion, and Section 4 includes the conclusion.

## 2. Methodology

**Figure 2:**
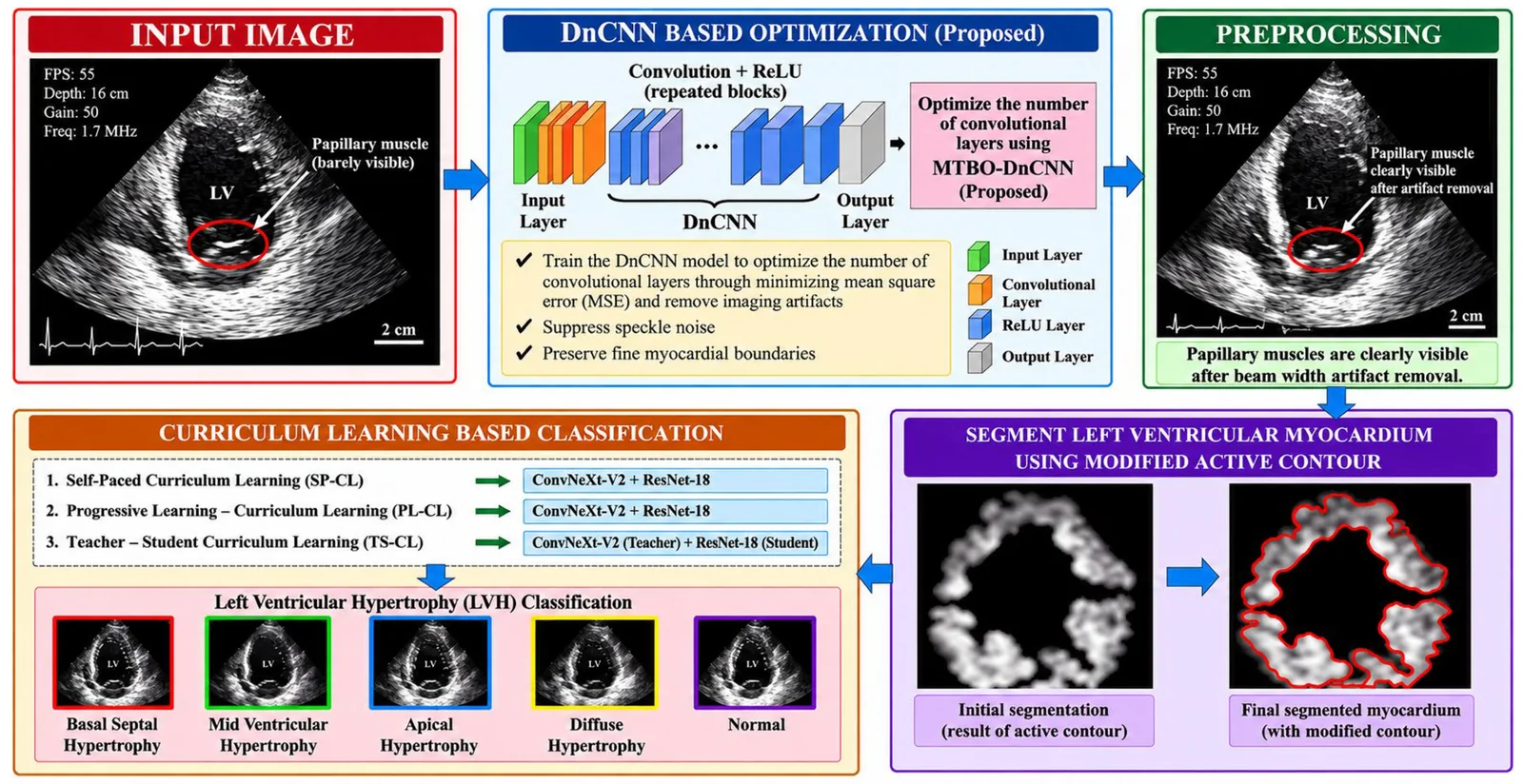
Proposed CDL model for left ventricular myocardial segmental classification.

The proposed curriculum-based deep learning (CDL) technique classifies the 2D short-axis (2D-SAX) echocardiographic views for left ventricular myocardial segmental diseases. The classification includes (i) BSH, (ii) MH, (iii) AH, and (iv) DH. The CDL approach is shown in Figure 2. In Figure 2, the architecture diagram explains that the proposed optimized MTBO-DnCNN reduces the rib and reverberation artifacts in the 2D echocardiographic image and enhances the image’s quality. Next, the MAC segmentation algorithm segments the image by evolving the initial contour through reducing the energies of the contours.

The left ventricular myocardial segmental area is classified using two convolutional deep learning (CDL) models: (i) ConvNeXt v2 and (ii) ResNet 18. The ConvNeXt v2 network integrates traditional convolutional networks with vision transformer models, enabling it to effectively extract spatial relationships among various left ventricular segments, such as the basal septum, mid-cavity, and apical segments. In contrast, the ResNet 18 model analyzes myocardial wall thicknesses and geometric changes in the myocardium by utilizing residual mapping from the input 2D echocardiographic images. This residual learning approach mitigates the vanishing gradient problem and enhances convergence speed. Additionally, a curriculum-based deep learning (CDL) framework is used to improve the classification of LVH patterns through three curriculum-learning strategies such as (i) Self-Paced Curriculum Learning (SP-CL), (ii) Progressive Learning–Curriculum Learning (PL-CL), and (iii) Teacher–Student Curriculum Learning (TS-CL). The retrospective study was conducted from April, 2024, to November, 2024, at M. George’s Bethlehem Ayurveda Specialty Hospital and Research Center, Vettimood, Kakkoor, Koothattukulam region, Ernakulam District, Kerala, India. It was performed in the Integrative Ayurvedic Cardiology Department combining traditional Ayurvedic diagnosis (like *Nadi Pariksha*/pulse diagnosis) with modern cardiac imaging tools. Ethical approval was obtained from IRB (approval number: 32689/CASR/2025/217, dated 8 December 2024). Written informed consent was obtained from all participants or their legal representatives.

### 2.1. Dataset Selection and Partitioning

A total of 2000 two-dimensional short-axis (2D-SAX) echocardiographic images were obtained from 200 patients. Images were selected based on acquisition, anatomical visibility, image quality, and clinical label criteria. Only images acquired at the mid-ventricular level with myocardial segments and clear endocardial and epicardial boundaries were used for this study. Images with severe blur, inadequate boundary visualization, missing or distorted myocardial segments, inappropriate acquisition planes, duplicate acquisitions, or unreliable clinical labels were excluded. The images were categorized into five classes: apical hypertrophy, basal septal hypertrophy, mid-ventricular hypertrophy, diffuse hypertrophy, and normal myocardium. A patient-level 80:20 split was performed, with 160 patients (1599 images) for training and 40 patients (401 images) for testing. The dataset was split at the patient level. All images were assigned to either the training or testing set. Thus, to prevent data leakage, no patient and no image from the same patient were present in both sets. Table 2 describes the dataset description, and inclusion and exclusion criteria for segmental LVH classification.

### 2.2. Annotation and Interobserver Agreement

Segmental LVH ground truth labels were independently assigned by two board-certified cardiologists with ≥10 years of echocardiographic experience, following the ASE/EACVI 17-segment model criteria. Disagreements were resolved by consensus with a third senior cardiologist. Annotation was performed using expert consensus, and the annotations of each cardiologist were blinded.

#### Interobserver Agreement

Interobserver agreement for segmental LVH classification between the two cardiologists was Cohen’s Kappa of κ = 0.88. Intraclass correlation coefficient for wall thickness measurement was ICC = 0.78.

### 2.3. Removal of Rib and Reverberation Artifacts in 2D Echocardiogram Using Proposed Mountaineering-Team-Based Deep Denoised Convolutional Neural Network

**Figure 3:**
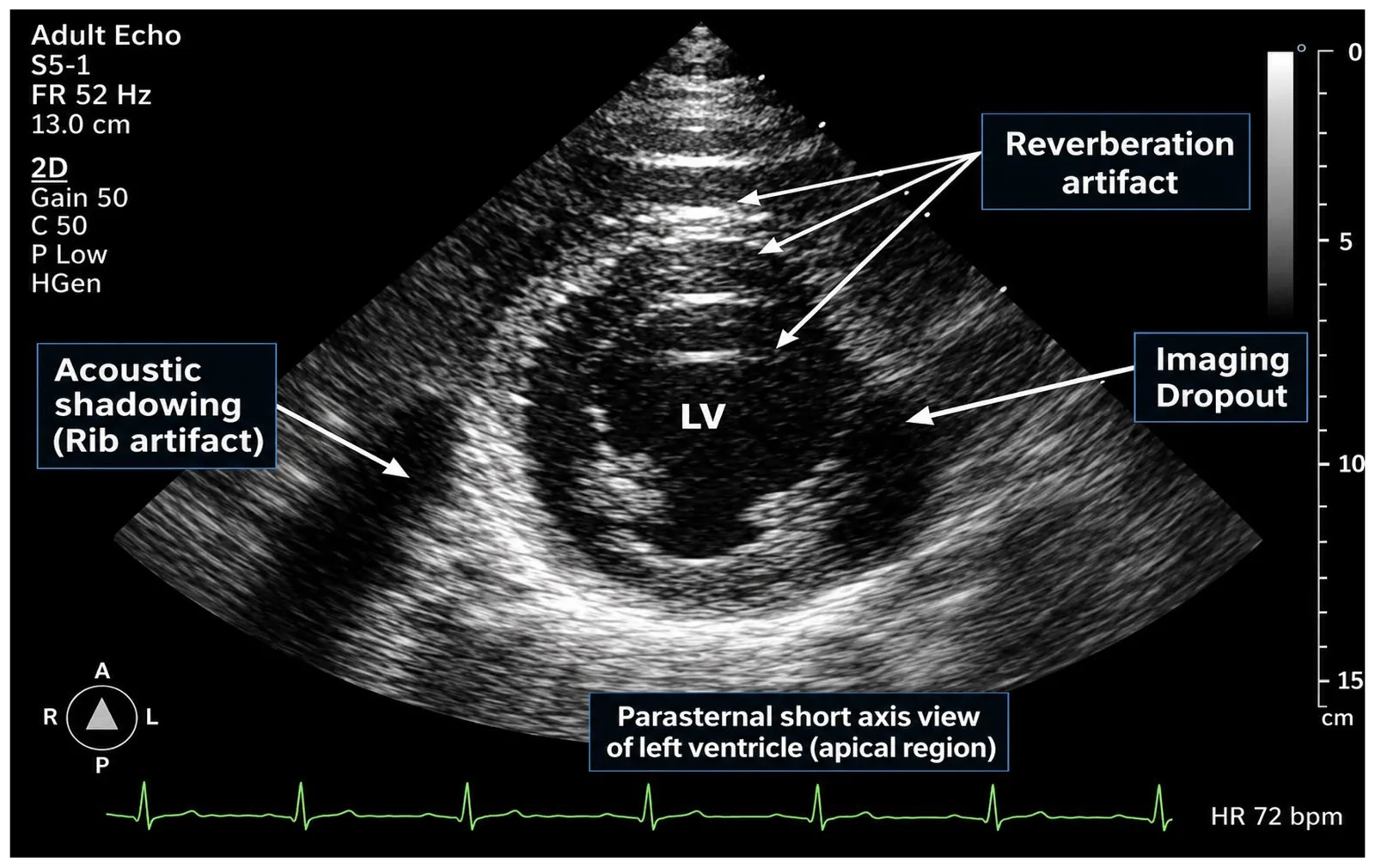
Artifacts in an echocardiogram [31]—rib artifacts occur due to heart movements from the transducer during respiration. Reverberation artifacts occur due to reflection. Dropout artifacts occur when the ultrasound waves are not penetrated properly in cardiac tissues. This leads to shadowing or dropout artifacts.

Imaging artifacts in 2D echocardiograms leads to misdiagnosis of left ventricular hypertrophy due to changes in myocardial boundaries. The artifacts occur due to physical movement of the patient and transducer pressure during ultrasound image acquisition. The artifacts in 2D echocardiogram are acoustic and shadowing artifacts, tissue reverberation artifacts, mirror images, and rib and beam-width artifacts. Among the above artifacts rib and reverberation artifacts reduction is a challenging task for researchers. Figure 3 shows the artifacts in a 2D echocardiogram.

The MTBO-DnCNN is used for reducing rib and reverberation artifacts from 2D echocardiographic images. Table 3 provides the architecture of MTBO-DnCNN.

Each convolutional layer in DnCNN is represented in Equation (1):

(1) $$O^{layer}=\sigma \left(w^{layer}*I^{layer-1}+b^{layer}\right)\;$$

In Equation (1), $O^{layer}$ refers to the output layer. $I^{layer-1}$ is the input image of the previous layer and $b^{layer}$ is the bias term of the previous layer. σ is the activation function.

The activation function learns the complex pattern from the LV myocardial structure. The activation function is represented in Equation (2):

(2) σ(x)=max(0,x)

In Equation (2), x is the input from the previous layer.

The batch normalization layer adjusts the mean and variance of the preceding input layer. It removes rib and reverberation artifacts from the left ventricular (LV) myocardium, which are caused by the effects of ultrasound waves. This reduction is achieved through enhanced gradient flow in the DnCNN network. The batch normalization layer is represented in Equation (3):

(3) $$F^{layer}=F^{layer}-\mu /\sqrt{\sigma^{2}+\epsilon }$$

In Equation (3), μ represents mean and σ represents variance.

The architecture contains more convolutional layers. The DnCNN architecture is computationally complex and takes longer to remove rib and reverberation artifacts from the LV 2D echocardiographic short-axis view. Therefore, MTBO-DnCNN optimizes the number of convolutional neural networks by minimizing the mean square error between the pixel intensities of the noisy input and the cleaned output images.

### 2.4. MTBO-DnCNN Algorithm for Artifact Removal in the Wall Regions

A mountaineering team consists of a number of mountaineers. The algorithm mimics the group of mountaineers climbing to reach the top of the mountain. The team follows the mountaineers’ movements and team coordination. Therefore, the MTBO algorithm optimizes the number of convolutional layers in the DnCNN network and eliminates rib and reverberation artifacts, improving the short-axis view of the left ventricular myocardium structure. A number of convolutional layers act as members of MTBO. Therefore, the number of convolutional layers is between the upper bound of 12 and the lower bound of four. Coordinated mountaineering selects the initial leader based on the highest number of convolutional layers. The remaining layers, such as the batch normalization layer and activation layer, act as team members. The new position of convolutional layer minimizes the mean square error between the original input and the denoised image. The other layer operations are moved towards the number of convolutional layers and reach the global optimum value. Equation (4) represents the new position.

(4) $${New\;position}_{conv}^{i}={Conv}_{i}+rand\times {Conv}_{leadr}-{Conv}_{i}$$

Hence, the convolutional layers are arranged from best to worst to minimize the mean square error and enhance the quality of the left ventricular myocardial structure for the classification of LVH. The new position of convolutional layers is shown in Equation (5).

(5) $${New\;position}_{conv}^{i}={Conv}_{i}+rand\times \left({Conv}_{leader}-{Conv}_{i}\right)+rand\times \left({Conv}_{individual}-{Conv}_{i}\right)\;$$

**Figure 4:**
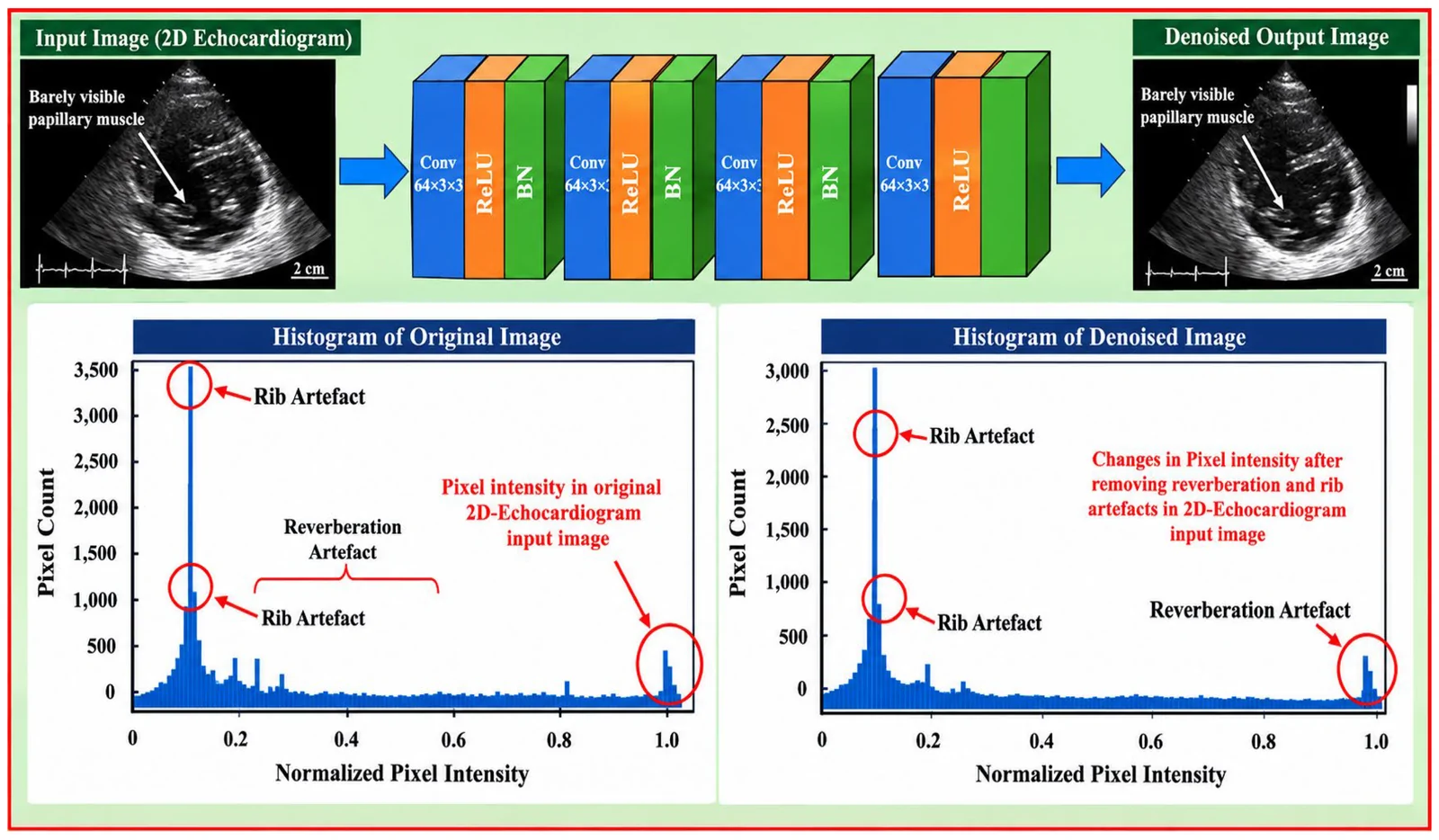
Rib and reverberation artiefacts removal using MTBO-DnCNN.

Figure 4 illustrates the removal of artifacts from a 2D EI image using MTBO-DnCNN. The histogram images depict the differences in pixel intensity during artifact removal. Table 4 presents a performance comparison of different network depths for MTBO-DnCNN. According to Table 4, the number of convolutional layers, ranging from five to 20, achieves a low mean squared error (MSE) of 0.000, a high peak in signal-to-noise ratio (PSNR) of 48.59, and a high structural similarity index (SSIM) of 0.99983. Based on this analysis, five convolutional layers are sufficient to effectively remove rib and reverberation artifacts from the 2D EI of the left ventricular myocardium.

### 2.5. Segmentation of Left Ventricular Myocardium Using Modified Active Contour Segmentation Algorithm

Segmental left ventricular hypertrophy (LVH) refers to hypertrophy localized to specific myocardial segments. To detect and classify segmental LVH, LV wall thickness is determined by measuring the distance between the endocardial and epicardial boundaries within each myocardial segment. The modified active contour (MAC) algorithm delineates the endocardial and epicardial boundaries from two-dimensional (2D) echocardiographic short-axis (SAX) images, enabling subsequent estimation of regional LV wall thickness. The active contour segmentation algorithm identifies the epicardial and endocardial boundaries of the left ventricular myocardium by evolving the initial contour toward the LV myocardial region. The algorithm begins with an initial contour placed inside the object and then moves towards the boundary by minimizing an energy function. The internal and external energy are defined in Equations (6) and (7).

(6) $${Energy}_{internal}=\frac{1}{2}\left[{elasticity\left|\frac{\partial v\left(s\right)}{\partial s}\right|}^{2}+{stiffness\left|\frac{\partial^{2}v\left(s\right)}{{\partial s}^{2}}\right|}^{2}\right]$$

(7) $${Energy\;}_{External}=-\left|gradient(Image(pixel\left(x,y\right))\right|$$

Total energy is defined in Equation (8) and total energy is defined as the sum of the internal and external energy.

(8) $$Total\;Energy{Energy}_{snake}={Energy\;}_{internal}+{Energy\;}_{external}$$

In Equation (9), negative sign indicates a high gradient area.

Energy minimization is defined in Equation (9).

(9) $$Contour\left(v\left(s\right)\right)={argmin}_{v}\left({Energy}_{snake}\right)$$

**Figure 5:**
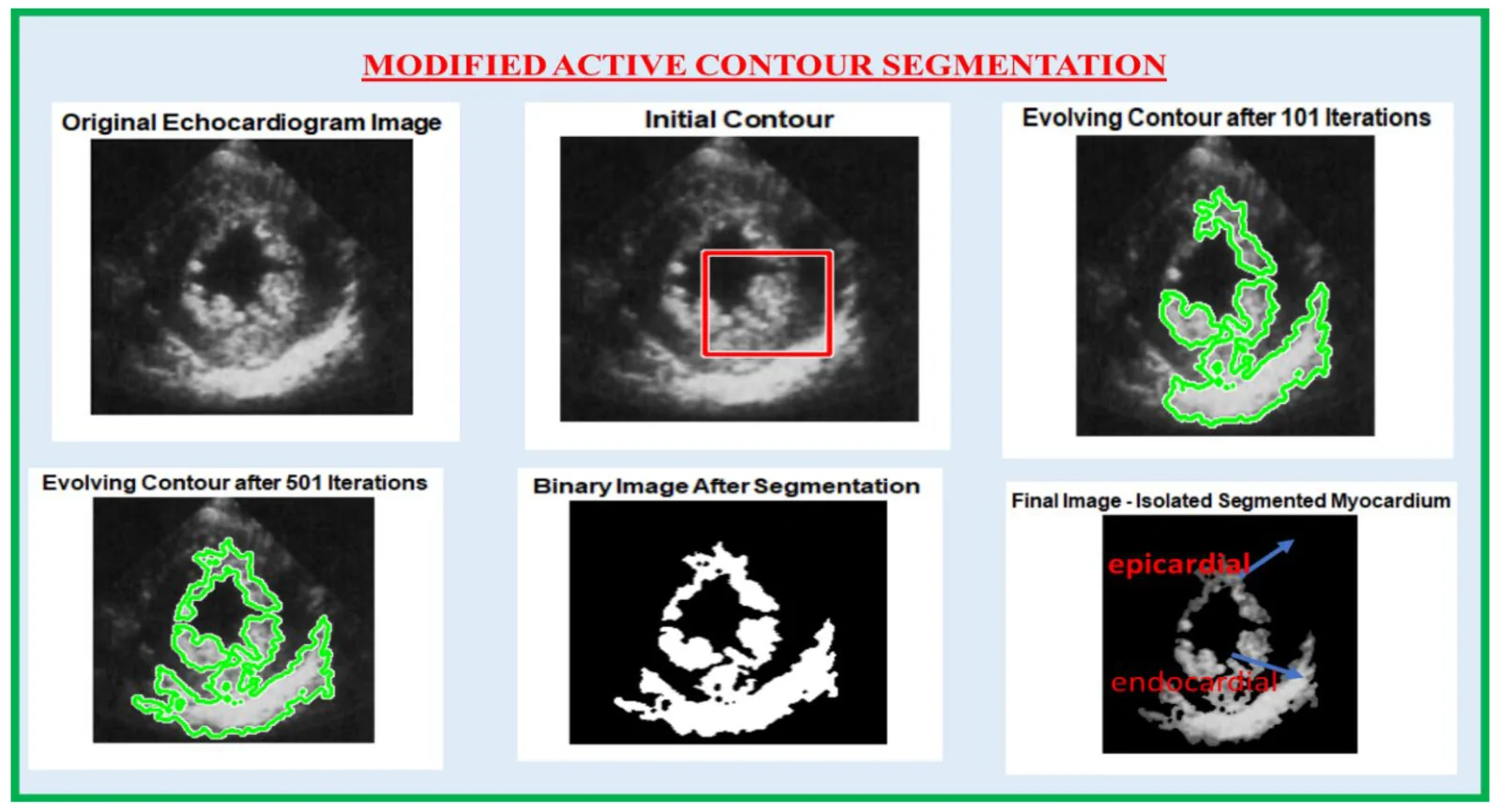
Segmentation of myocardial structure using modified active contour segmentation.

**Figure 6:**
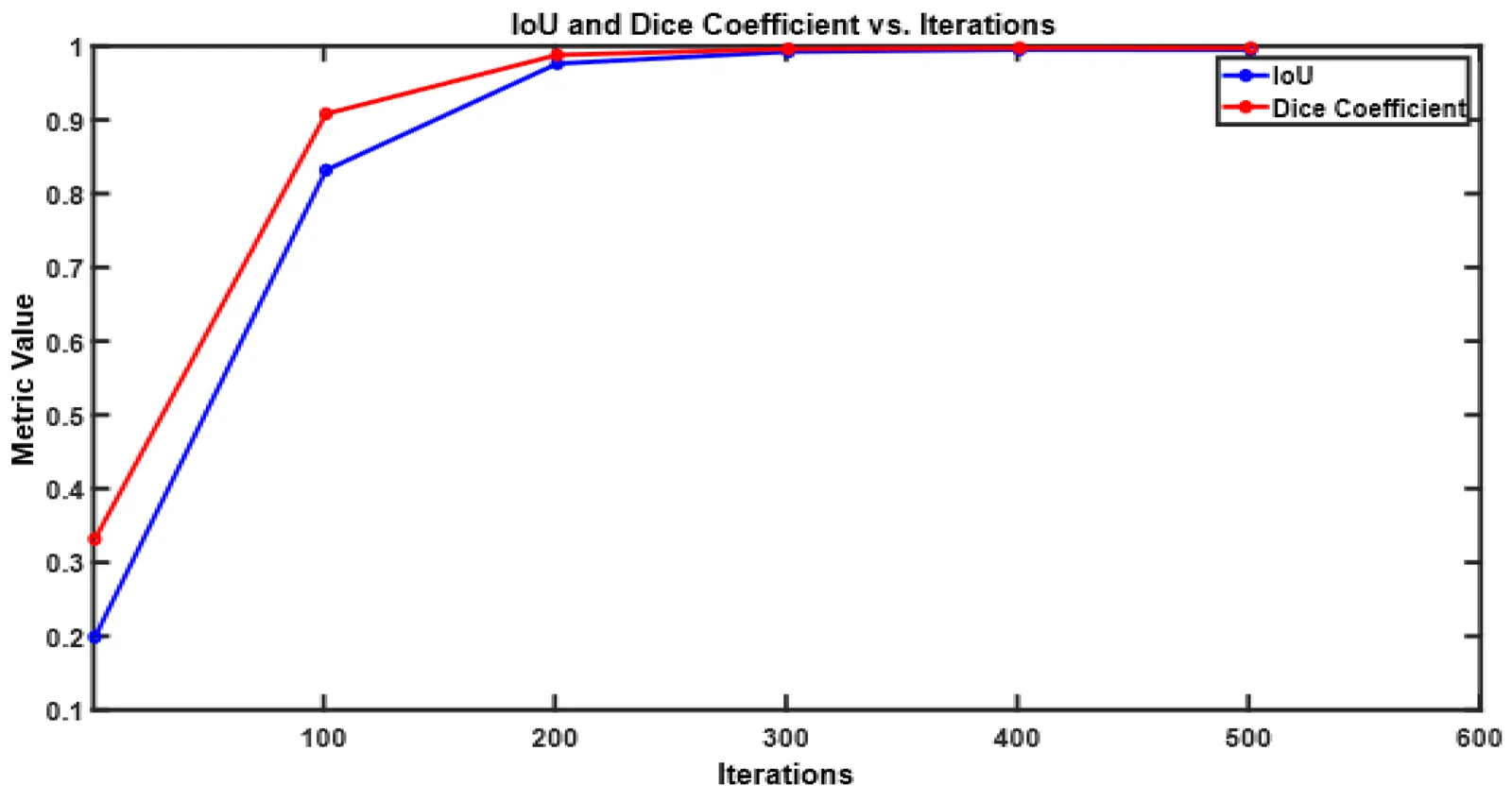
Comparison of IoU and dice in LVH myocardium.

The segmentation of the left ventricular myocardial structure is illustrated in Figure 5. This figure displays the enhanced myocardial image, the evolution of the contour, the binary image, and the segmented myocardial structure. Initially, the square contour is configured as a rectangular contour with the values [80, 150, 80, 150]. Over 600 iterations, the contour progressively expands toward the epicardium and endocardium boundaries of the left ventricle myocardium. The MAC algorithm delineates the boundaries of the left ventricular myocardium. Table 5 and Figure 6 show the performance analysis of MAC to delineate the myocardium boundaries.

### 2.6. Proposed Architecture of Curriculum-Based Deep Learning (CDL) Model

**Figure 7:**
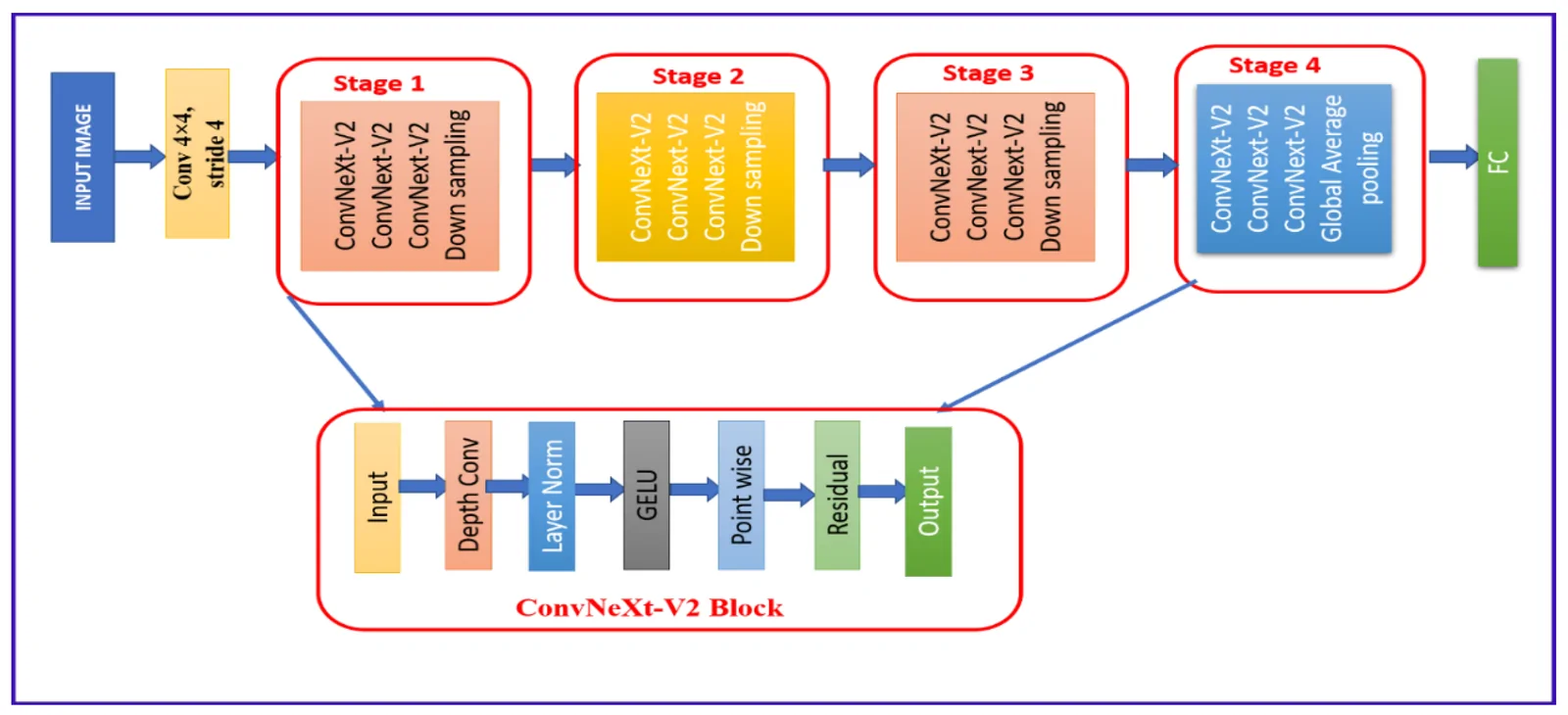
Architecture of ConvNeXt-V2 model for LVH classification.

Traditional deep learning image classification models face several challenges: (i) they require large labeled datasets; (ii) they demand significant computational time for training; (iii) they necessitate extensive hyperparameter tuning; (iv) they require domain expertise for feature extraction; (v) they are prone to overfitting with small datasets. ConvNeXt-V2 addresses these limitations through a masked autoencoder with self-supervised learning. This masked autoencoder uses sparse pixel arrays and preserves detailed information about the 2D segmented left ventricular myocardium structure, effectively overcoming the drawbacks associated with traditional models. Figure 7 illustrates the architecture of the ConvNeXt-V2 model applied to LVH classification.

The ConvNeXt-V2 model effectively extracts features from segmented myocardial structures, including the basal anteroseptal, basal inferoseptal, mid-anteroseptal, mid-inferoseptal, basal anterolateral, and basal inferolateral regions. In myocardial segmentation, features such as the basal anteroseptal and basal inferoseptal often overlap, leading to redundancy in the feature maps due to the convolutional layers. Traditional deep learning models suffer from feature collapse, which contributes to misdiagnosis in LVH classification.

To address these issues, ConvNeXt-V2 incorporates Global Response Normalization (GRN). GRN employs three key techniques: (i) aggregating global features, (ii) normalizing these features, and (iii) calibrating features to reduce redundancy and improve feature extraction accuracy. This approach mitigates feature collapse and enhances the reliability of LVH classification.

**Figure 8:**
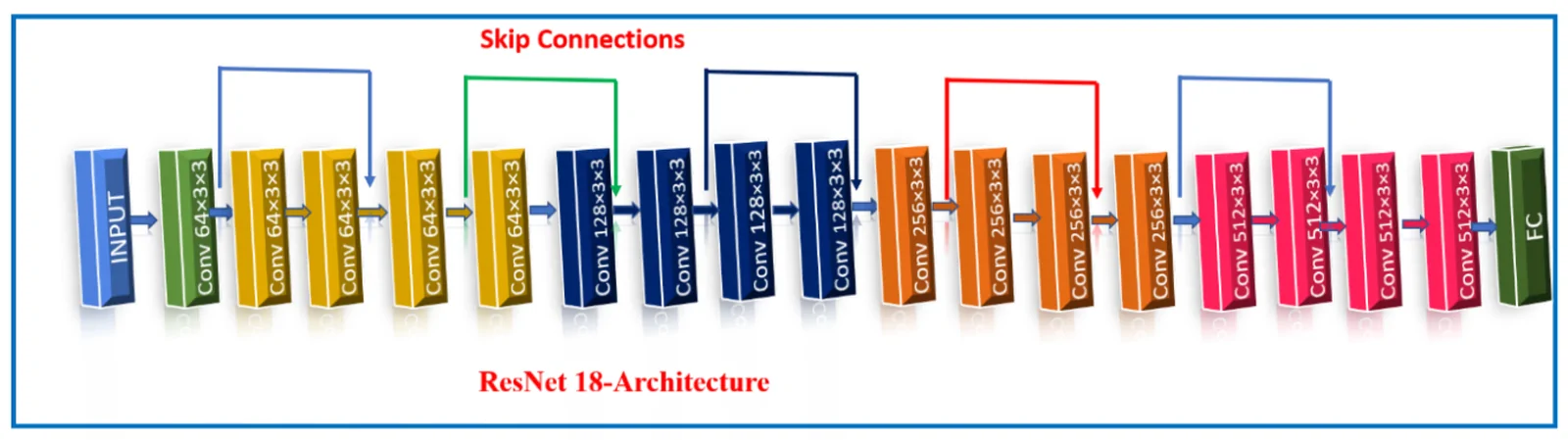
Architecture of ResNet 18 model for LVH classification.

The ResNet-18 model addresses the vanishing gradient problem through learning residual functions and skip connections as shown in Figure 8, which facilitates gradient flow and accelerates the training process. Table 6 shows the comparison of feature extraction between traditional ResNet18 deep learning model and ConvNeXt-V2 model.

Curriculum learning introduces training samples according to their learning difficulty and enhance model generalization, mitigate overfitting, improve feature representation learning, and accelerate convergence. In this study, ConvNeXt-V2 and ResNet-18 are trained using three curriculum-learning strategies: Self-Paced Curriculum Learning (SP-CL), Progressive–Curriculum Learning (PL-CL), and Teacher–Student Curriculum Learning (TS-CL). Table 7 compares the performance of these strategies for segmental LVH classification.

### 2.7. Hyperparameter Optimization and Model Training

The LVH-DL framework’s hyperparameters are divided into preprocessing, segmentation, and classification. The MTBO-DnCNN module’s convolutional layers were optimized from 4 to 12 over 10 epochs. The optimal configuration was five 3 × 3 convolutional layers, each with 64 feature channels, ReLU activation, and batch normalization. Myocardial segmentation, performed by the MAC algorithm, used 300 iterations, a 5 × 5 Gaussian smoothing filter, a Sobel gradient operator, and specific contour parameters. Segmentation performance was evaluated using the dice coefficient and IoU. For classification, ConvNeXt-V2 was trained with the Adam optimizer, an initial learning rate of 1 × 10^−4^, a batch size of 16, and cross-entropy loss. Cosine annealing managed learning-rate scheduling. The baseline ConvNeXt-V2 model trained for 30 epochs.

Curriculum learning involved Self-Paced Curriculum Learning (SP-CL), Progressive Curriculum Learning (P–CL), and Teacher–Student Curriculum Learning (TS-CL). SP-CL used an initial loss threshold of 0.5, increasing by 0.1 per epoch. PCL began with 30% of the easiest samples, increasing the fraction by 10% per epoch. For TS-CL, sample difficulty was based on 1:1 teacher prediction confidence with a 0.7 difficulty threshold. The teacher network was frozen during student training, while the ConvNeXt-V2 student network remained trainable. The model with the best validation performance was selected for final testing. A comprehensive summary of all hyperparameters and implementation settings is provided in Table 8 for reproducibility.

## 3. Results and Discussions

The dataset comprises 2000 images of patients aged between 35 and 80 years. The images were acquired at the mid-ventricular level using a GE Vivid ultrasound machine. Each image is captured at a resolution of 512 × 512 pixels, featuring six standard myocardial segments based on the 17-segment model. The dataset includes patients who have been clinically confirmed to have left ventricular hypertrophy (LVH) through echocardiography and ECG. Patients with congenital defects or arrhythmias were excluded due to structural abnormalities and irregular myocardial motion in echocardiographic imaging, which can affect the accurate delineation of myocardial segments.

LV wall thickness measurement and myocardial boundary delineation to evaluate the reliability and reproducibility of the annotations are shown in Table 9.

Hence, the classification of left ventricular hypertrophy is analyzed through the following metrics such as precision, recall, F1-score, sensitivity, specificity, accuracy and receiver operating characteristics (ROC) curve. The metrics are represented in Equations (10)–(15).

(10) Recall=TP/(TP+FN)

(11) Precision=TP/(TP+FP)

(12) F1−Score=(2×precision×recall)/(precision+recall)

(13) Specificity=TN(TN+FP)

(14) Accuracy=(TP+TN)(TP+TN+FP+FN)

TP, TN, FP, and FN are represented as follows:

TP = The model correctly classifies presence of LVH;

FP = The model incorrectly classifies presence of LVH;

TN = The model correctly classifies the absence of LVH;

FN = The model incorrectly classifies the absence of LVH.

Moreover, curriculum-based deep learning models are analyzed through the following metrics such as convergence rate, class imbalance metrics Gini and entropy, gradient norm, and correlation matrix.

Convergence Rate = (Loss(t − 1) − Loss(t))/Loss(t − 1)(15)

In Equation (15), the loss at time step t is denoted, and the loss at the next time step is also specified. Therefore, the convergence rate is defined by how quickly the model’s loss function approaches its minimum value. A higher convergence rate indicates a more complex curriculum learning model.

(16) $$Gini\;index=1-\sum_{i=1}^{n}{Proption\;of\;instance\;to\;the\;class}_{i}^{2}$$

In Equation (16), n indicates the number of classes. A Gini index close to zero signifies that there is no class imbalance in the particular learning strategy, while a Gini index close to one indicates class imbalance in the curriculum learning strategy.

To assess the easier and harder tasks in the curriculum learning strategy, the gradient norm is used. A high gradient norm indicates that the model takes large steps during training, which can lead to instability. Conversely, a lower gradient norm results in slower learning during the training process of the proposed model.

(17) $$Gradient\;norm={\left\|∆L(\theta )\right\|}_{2}$$

In Equation (17), L(θ) is the gradient of loss function.

### 3.1. ResNet 18-Based Curriculum Learning for LVH Classification

**Figure 9:**
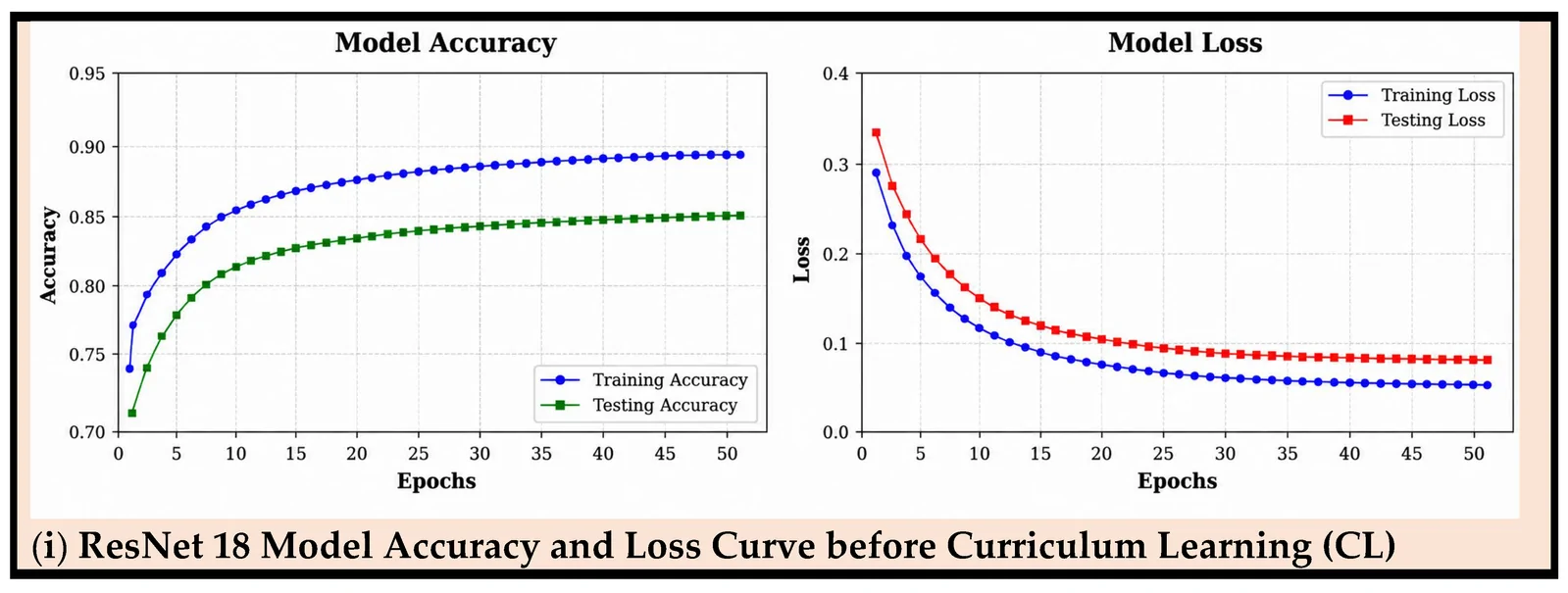

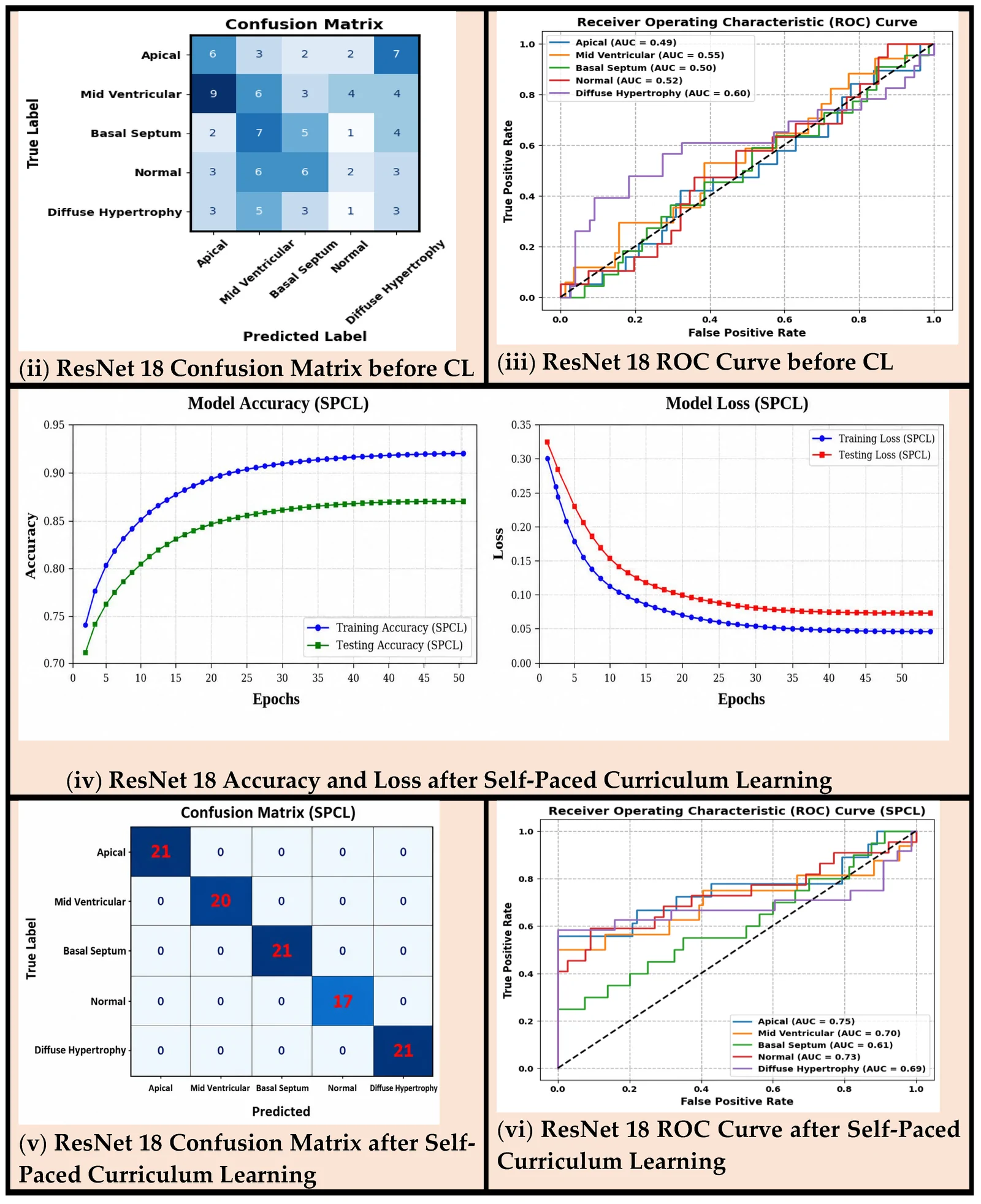
Performance analysis of LVH classification using ResNet-18 before CL and after CL.

Figure 9 presents the performance analysis of left ventricular hypertrophy (LVH) classification using the ResNet-18 model, both before and after the Self-Paced Curriculum Learning (SP-CL). Segmental LVH subtypes such as apical and mid-ventricular hypertrophies, exhibit similar patterns due to thrombus formation and diastolic function. These subtypes are indistinguishable which lead to misdiagnosis in LVH classification. The ResNet-18 architecture classifies the complex echocardiographic patterns of segmental LVH. The model achieved an accuracy of about 89% between similar LVH subtypes in Figure 9i,iii. The limited depth of ResNet-18 results in overfitting imbalanced data and sensitivity to hyperparameters, which lowers the area under the ROC curve, as depicted in Figure 9iii. The ROC values for apical, mid-ventricular, and basal septum subtypes are all 0.5, reflecting the model’s inability to effectively discriminate between positive and negative LVH samples.

To address these shortcomings, SP-CL was applied to the ResNet-18 model. SP-CL gradually introduces training data from simpler to more complex cases, stabilizing the loss and accuracy. This adjustment improves training and testing accuracies to approximately 90% and 87, as shown in Figure 9iv,v. Furthermore, the SP-CL ResNet-18 increases the area under the ROC curve for apical (0.75), mid-ventricular (0.7), basal septum (0.67), and diffuse hypertrophy (0.69) subtypes, as illustrated in Figure 9vi. This moderate improvement in classification performance highlights SP-CL’s ability to adaptively adjust the learning rate allowing for better discrimination of positive and negative instances in segmental LVH classification.

**Figure 10:**
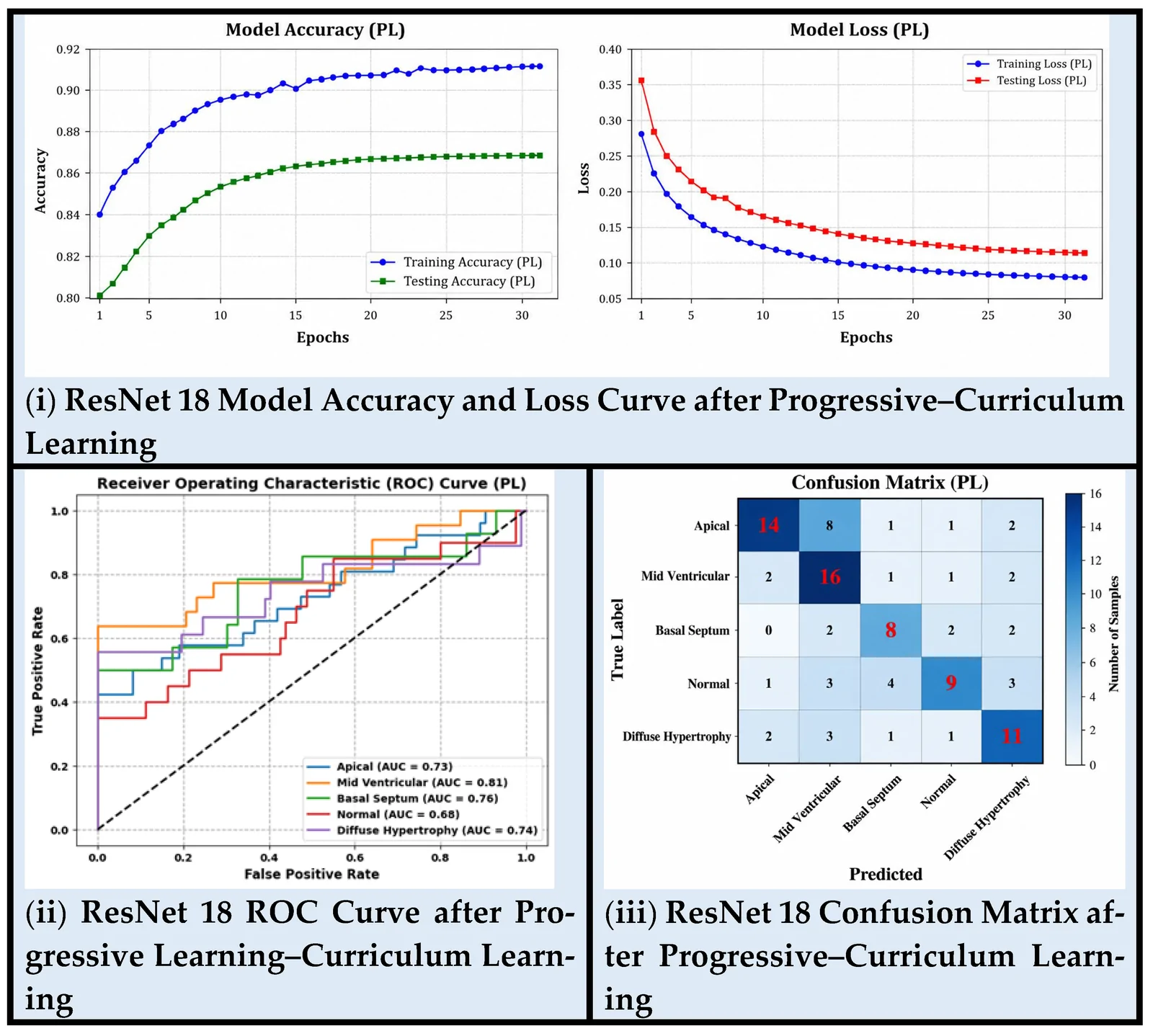
Performance analysis of LVH classification using P–CL ResNet-18.

Figure 10 presents the performance analysis of the Progressive–Curriculum Learning (P–CL)-based ResNet-18 model. The standard Self-Paced Curriculum Learning (SP-CL) ResNet-18 model has several limitations, including reliance on a predefined curriculum, an inability to capture complex features essential for learning similar segmental left ventricular hypertrophy (LVH) patterns such as apical, basal septum, and mid-ventricular hypertrophies, and longer training times; The SP-CL ResNet-18 model provides moderate discrimination in LVH classification.

In contrast, The P-CL ResNet-18 model adjusts the selection of training examples according to their learning difficulty, and faster convergence and more efficient learning. This adaptive approach enhances performance, resulting in training and testing accuracies of 93.5% and 89.5%, respectively, as shown in Figure 10i,iii. The area under the ROC curve for segmental LVH classification ranges from 0.73 to 0.81, as depicted in Figure 10ii. These improvements show the P–CL ResNet-18 model’s enhanced capability to classify segmental LVH patterns effectively.

**Figure 11:**
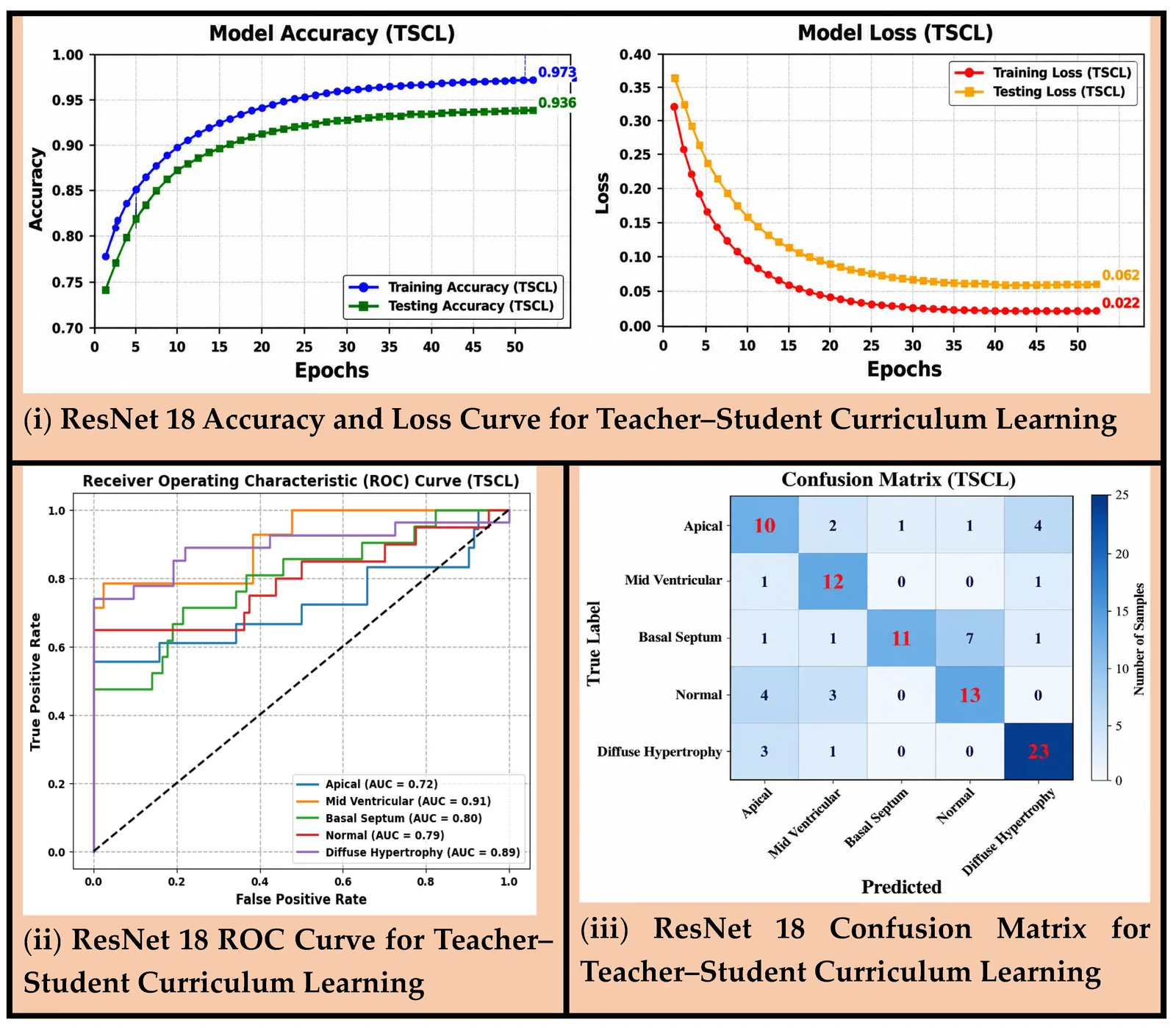
Performance analysis of LVH classification using TS-CL ResNet-18.

Figure 11 illustrates the performance analysis of left ventricular hypertrophy (LVH) classification using Teacher–Student Curriculum Learning (TS-CL) with the ResNet-18 model. In TS-CL, the teacher dynamically adjusts the curriculum based on the student’s learning progress, leading to improved training and testing accuracies of 95% and 91.8% as shown in Figure 11i,ii. In contrast, Self-Paced Curriculum Learning (SP-CL) uses a fixed curriculum, which limits classification performance due to unstructured design. Progressive–Curriculum Learning (PL–CL) effectively handles data at various levels of complexity, yet increases computational demands due to uncontrollable curriculum adjustments.

TS-CL ResNet-18 demonstrates superior classification performance, with enhanced discrimination between positive and negative samples in segmental LVH classification. TS-CL achieves an area under the ROC curve (AUC) of 0.91 for mid-ventricular hypertrophy and 0.89 for diffuse hypertrophy, as shown in Figure 11iii. This shows the model’s robust classification capabilities.

Table 10 shows the comparative analysis of different curriculum learning (CL) strategies in ResNet-18 for left ventricular hypertrophy (LVH) classification. Self-Paced Curriculum Learning (SP-CL) demonstrates moderate convergence and increases the Gini index to 0.32, indicating a reduction in class imbalance. However, this higher Gini index negatively impacts LVH classification accuracy. SP-CL generates high gradient loss when selecting varying levels in the curriculum. Progressive–Curriculum Learning (P–CL), converges more quickly due to its ability for dynamic adjustment in the curriculum during runtime. P–CL shows a lower Gini index compared to SP-CL, thereby improving class imbalance handling. P–CL produces lower gradient values, allowing for a gradual adjustment in curriculum during ResNet-18 training for LVH classification. Teacher–Student Curriculum Learning (TS-CL) achieves the fastest convergence, benefiting from the teacher’s ability to adjust the curriculum throughout the training process. TS-CL further reduces the Gini index, indicating highly balanced classes during training. The gradient norm is minimized, as the structured curriculum guided by the teacher leads to more efficient training of the ResNet-18 model for LVH classification.

### 3.2. Analysis of ConvNeXt-V2 Curriculum-Based LVH Classification

Table 11 shows the performance analysis of left ventricular hypertrophy (LVH) classification using the ConvNeXt-V2 model with Self-Paced Curriculum Learning (SP-CL), Progressive Learning–Curriculum Learning (P–CL), and Teacher–Student Curriculum Learning (TS-CL). Among these, TS-CL demonstrates faster convergence due to dynamic curriculum selection, outperforming SP-CL and P–CL. ConvNeXt-V2’s architecture includes a Global Response Normalization (GRN) layer, which scales channel activation based on the relative magnitude of activation maps, thereby preventing feature collapse. Additionally, the model incorporates a masked autoencoder that learns features from masked images, enhancing the model’s flexibility across different curriculum learning strategies for LVH classification.

ConvNeXt-V2 with TS-CL shows superior performance in segmental LVH classification compared to ResNet-18. Apical hypertrophy achieves the highest recognition accuracy, as it appears spade-like in short-axis echocardiographic views, while the mid-ventricular and basal walls remain relatively normal. This enables the model to effectively distinguish complex LVH features. However, the mid-ventricular septum and basal septum exhibit increased wall thickness due to diastolic dysfunction and hypertension, which causes these hypertrophies to share structural similarities, thereby reducing classification performance in these regions.

Table 12 shows the performance comparison of various learning strategies in segmental left ventricular hypertrophy (LVH) classification using the ConvNeXt-V2 model. ConvNeXt-V2 applies layer normalization and utilizes large kernel sizes in the initial convolutional layers, enhancing LVH classification by computing the mean and variance of each image across layers. These large kernels are particularly effective in capturing complex features from segmental LVH subtypes such as apical and mid-ventricular hypertrophies, which often appear similar in 2D echocardiograms during left ventricular diastolic function. Diastolic function preserves the ventricular size during diastole, allowing the ConvNeXt-V2 model to capture long-range dependencies through depth-wise convolution and initial large convolutional layers.

To further enhance ConvNeXt-V2’s performance in LVH classification, Teacher–Student Curriculum Learning (TS-CL) is applied. TS-CL accelerates convergence, reducing the number of epochs required for training. Additionally, the Gini index reflects minimized inequality in training samples, improving performance compared to Progressive–Curriculum Learning (P–CL) and Self-Paced Curriculum Learning (SP-CL).

Table 13 presents a class-wise performance comparison between ResNet-18 and ConvNeXt-V2 for segmental LVH classification. ConvNeXt-V2 outperforms ResNet-18 across all five classes in accuracy, precision, recall, F1-score, and AUC. ConvNeXt-V2 improves the macro-average accuracy from 93.20% to 96.10% and the macro-average AUC from 94.24% to 96.94%. The McNemar and DeLong *p*-values are below 0.05, indicating statistically significant differences between the two models.

## 4. Discussion

Traditionally, left ventricular hypertrophy (LVH) is diagnosed through manual measurement, i.e., measuring the thickness of the left ventricular wall in specific segmental regions, such as the apical or basal areas. The measurement of left ventricular wall thickness (LVW) is typically performed using the following Equation (18).

(18) $$LVM=0.8\times 1.04\;\;\;\;\;\;\;\;\;\;\;\;\;\;\;\;\;\;\times {\left(intraventricular\;septal\;thickness\;\;\;\;\;\;\;\;+left\;ventricular\;end\;diastolic\;function\;\;\;\;\;\;\;\;\;+posterior\;wall\;thickness\;at\;end\;diastole\right)}^{3}\;\;\;\;\;\;\;\;\;\;-{left\;ventricular\;end\;diasolic\;function}^{3}+0.6\;$$

Moreover, LVMI is calculated through body surface area using Mosteller Formula. Hence, LVMI is defined in Equation (19).

(19) LVMI=LVM/BSA

**Figure 12:**
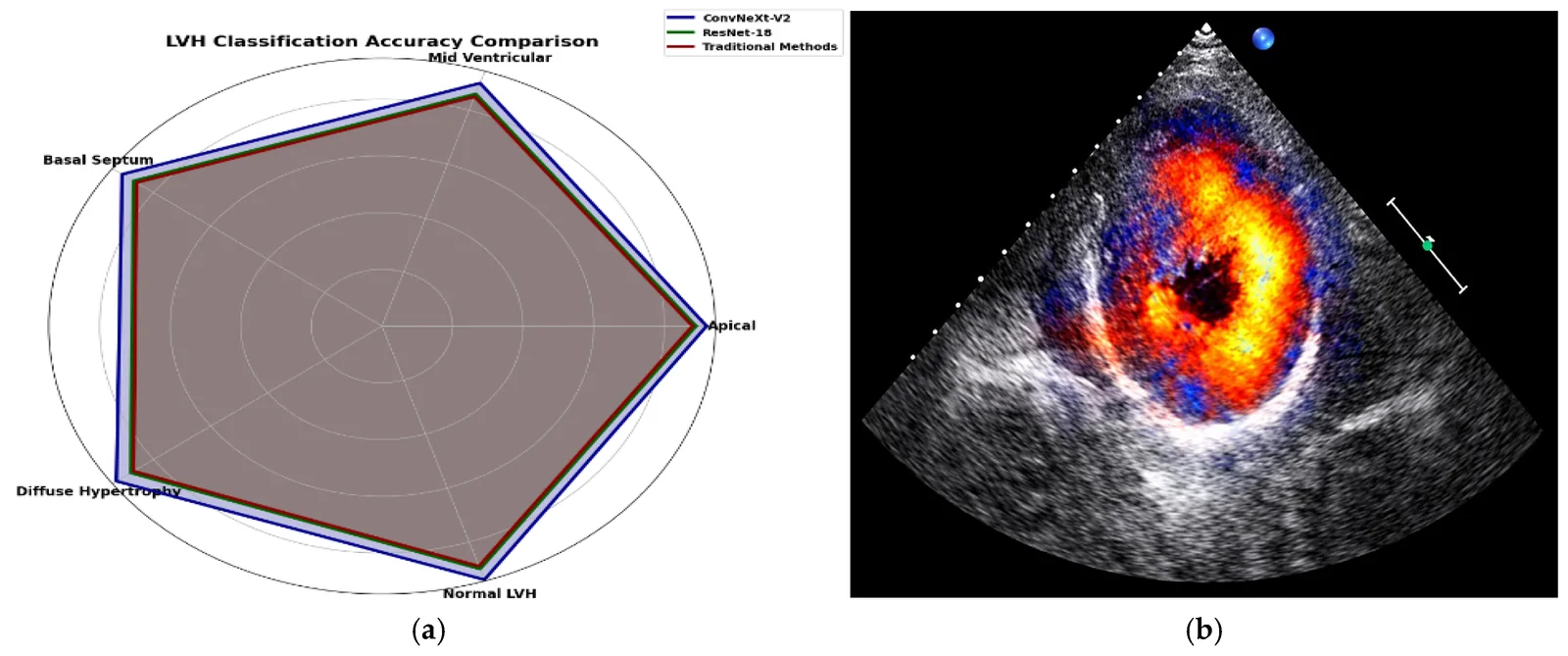
(**a**) Comparison of traditional and proposed CL–deep learning Architecture in LVH classification. (**b**) Grad CAM visualization of apical hypertrophy. Color Doppler overlay indicates blood-flow direction and velocity, with red/orange and blue regions representing flow toward and away from the transducer, respectively; color intensity reflects the relative magnitude of flow velocity.

Classifying left ventricular hypertrophy (LVH) using left ventricular mass index (LVMI) is challenging due to the inaccuracies in measuring body surface area (BSA), which is population-specific and may not accurately reflect individual variations. This often results in misclassification of LVH. To address this issue, the present study proposes segmental LVH classification by analyzing 2D echocardiographic short-axis (SAX) view using curriculum-based deep learning architectures. While ResNet-18 struggles to capture complex patterns among similar segmental hypertrophies such as apical and mid-ventricular types, the ConvNeXt-V2 model effectively captures long-range dependencies within segmental LVH. Consequently, the TS-CL-based ConvNeXt-V2 demonstrates superior classification accuracy compared to traditional methods and the ResNet-18 architecture. Figure 12 depicts the comparative performances of LVH classification between traditional methods and the proposed curriculum-based deep learning architectures. Figure 12a shows that ConvNeXt-V2 outperforms in segmental LVH classification compared to ResNet-18 and traditional methods. Moreover, ConvNeXt-V2 captures the long-range inter-segmental dependencies within the myocardial regions. The Grad-CAM visualization in Figure 12b shows that the highest activation intensity is indicated in the red and yellow regions, indicating increased wall thickness in the apical myocardial region. Table 14 shows the comparison between experienced echocardiographers, with agreement across all LVH categories. The proposed LVH-DL model achieved accuracies ranging from 95.0% to 97.2%, and agreement with expert annotations ranged from 93.6% to 97.1%. Cohen’s κ values of 0.85–0.91 indicate almost-perfect agreement with the expert reference standard. The highest agreement was observed for normal myocardium and diffuse hypertrophy, whereas basal septal hypertrophy showed comparatively lower agreement.

This study evaluated a deep learning framework (LVH-DL) for classifying segmental left ventricular hypertrophy (LVH) using two-dimensional (2D) short-axis echocardiography. The framework integrates MTBO-DnCNN for artifact suppression, modified active contour segmentation (MACS) for myocardial boundary delineation, and a curriculum-based ConvNeXt-V2 classifier for segmental LVH classification. The framework achieved class-specific accuracies of 96.5% for apical hypertrophy, 95.0% for basal septal hypertrophy, 95.8% for mid-ventricular hypertrophy, and 97.2% for diffuse hypertrophy, demonstrating its capability to identify distinct segmental LVH patterns through an integrated deep learning pipeline.

Interpretation of Key Findings

High classification accuracy was achieved despite challenges from rib and reverberation artifacts. The MTBO-DnCNN preprocessing stage yielded a peak signal-to-noise ratio (PSNR) of 48.59 dB and a structural similarity index (SSIM) of 0.99983, indicating lossless artifact suppression and preservation of left ventricular wall structure. This step is crucial as artifacts increase misclassification risk in previous echocardiography models. The MACS algorithm accurately identified endocardial and epicardial boundaries across segmental regions.

ConvNeXt-V2 outperformed ResNet-18, likely due to its Global Response Normalization (GRN) mechanism, which mitigates feature collapse from overlapping segmental patterns. Curriculum-based learning strategies (SP-CL, PL–CL, and TS-CL) improved generalizability by introducing complex training samples, reducing overfitting on imbalanced datasets, and stabilizing gradient norms.

Comparison with Existing Literature Studies

Previous AI approaches to LVH focused on global detection, structural abnormality screening, or etiological differentiation, not anatomical segmental classification. ECG-based models lack spatial resolution for localizing hypertrophy to specific segments. Deep learning models for chest radiography detect severe global LVH but lack anatomical granularity for segmental classification.

Echocardiography-based deep learning models analyze global LV structure and function, not regional hypertrophy patterns. Radiomics studies combined with deep transfer learning differentiate hypertrophic cardiomyopathy (HCM) etiologies but address the cause, not anatomical distribution. This study addresses this gap by classifying specific myocardial regions, aiding treatment planning and risk stratification.

Furthermore, previous segmentation models (e.g., U-Net, Inception ResNet-v2) for echocardiography struggled with inaccurate border delineation in heterogeneous hypertrophy. The proposed MACS algorithm, using energy-minimization-based contour evolution, achieves more precise boundary detection than fixed-architecture segmentation models.

Unexpected Findings and Limitations

Diffuse hypertrophy achieved the highest accuracy (97.2%). Globally thickened walls in diffuse patterns produced uniform and distinct feature signatures, reducing inter-class confusion. In contrast, basal septal hypertrophy had the lowest accuracy (95.0%), reflecting spatial overlap and ambiguity with mid-ventricular wall features in short-axis views.

Study limitations include a single-center dataset from one GE Vivid ultrasound machine at a fixed imaging plane, limiting generalizability across different systems, operators, and patient populations. Excluding patients with congenital defects or arrhythmias restricts applicability. The framework was validated only for 2D short-axis echocardiographic images. Integrating complementary views (e.g., apical four-chamber, parasternal long-axis) could improve accuracy for basal and apical subtypes. Multicenter studies are needed to confirm robustness across diverse imaging protocols.

Clinical Implications and Future Directions

The LVH-DL framework automates LVH classification in clinical echocardiography, providing cardiologists with information to assess hypertrophy severity and distinguish conditions like hypertensive heart disease, hypertrophic cardiomyopathy, and athlete’s heart. Early and accurate identification guides therapeutic decisions, including antihypertensive therapy, implantable devices, and surgical myectomy.

Future research should prioritize multicenter validation across diverse ultrasound platforms and patient demographics. Integration of multi-view echocardiographic data, longitudinal follow-up, and explainability methods are recommended. Extending the framework to real-time clinical deployment via edge-computing platforms and translation of findings into bedside diagnostic support tools is also crucial.

The small dataset size (2000 images) leads to overfitting in deep-learning-based segmental LVH classification. However, the use of curriculum learning (CL) strategies, such as SP-CL, P–CL, and TS-CL, mitigates overfitting by dynamically selecting training samples based on the student’s performance in TS-CL. This approach achieves the lowest Gini index (0.24), minimal gradient norm (3.9), and the highest classification performance across all LVH subtypes. Therefore, curriculum learning not only enhances convergence and classification accuracy but also serves as a form of implicit regularization, ensuring better generalization in small-scale echocardiographic datasets. Table 15 shows calibration performance analysis of the TS-CL ConvNeXt-V2 model.

**Figure 13:**
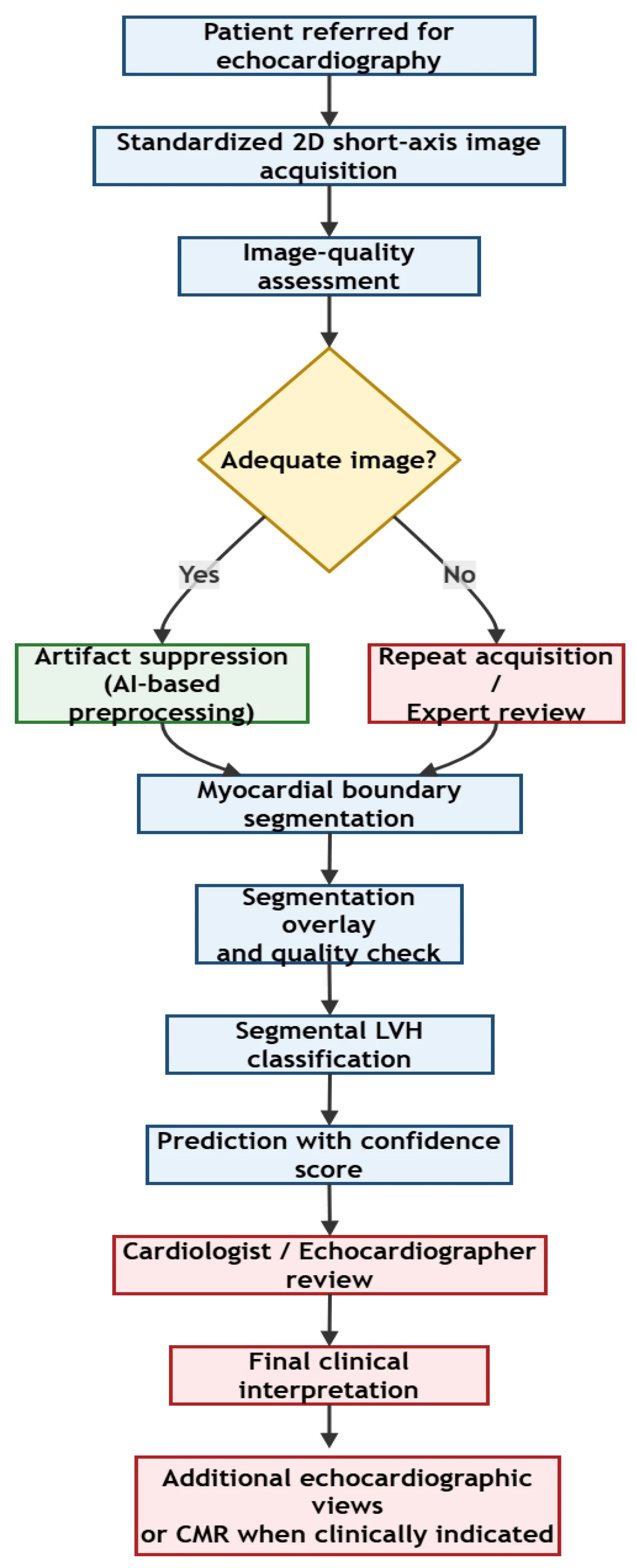
Clinical deployment of the proposed LVH-DL framework for segmental LVH classification.

The clinical applicability of the proposed LVH-DL framework provides specific segmental LVH classification. Apical hypertrophy directs clinicians toward hypertrophic cardiomyopathy workup and anticoagulation treatment, basal septal hypertrophy toward outflow tract obstruction management, mid-ventricular hypertrophy toward cavity obstruction and storage disorder screening, and diffuse hypertrophy toward cardiac amyloidosis exclusion and athlete’s heart differentiation. The automated end-to-end pipeline eliminates operator-dependent variability in wall thickness measurement and boundary delineation. However, the clinical translation of this framework is limited due to its single-center validation, absence of external multicenter testing, and exclusion of patients with arrhythmias and congenital defects. Prospective multicenter validation across diverse ultrasound platforms, patient demographics, and clinical settings and evaluation using FDA software as medical device guidelines or EU MDR standards remain essential prerequisites before this framework can be deployed as a point-of-care diagnostic support tool in routine clinical echocardiography practice. Figure 13 shows clinical deployment of the proposed LVH-DL framework for segmental LVH classification.

### 4.1. Limitations

The study was conducted using a single-center dataset, which limits generalizability and reproducibility across different institutions, imaging protocols, and ultrasound vendors. Multicenter validation is necessary to ensure the robustness and stability of the proposed model in diverse clinical environments. Furthermore, the framework was evaluated using short-axis (SAX) echocardiographic views. Although the SAX view provides optimal visualization of segmental myocardial wall thickening, incorporating additional views, such as the parasternal long-axis (PLAX), apical four-chamber (A4C), and apical two-chamber (A2C) views, provide a more comprehensive assessment of LV anatomy. Finally, while the proposed optimization strategy improves segmentation and classification performance, integration of clinically guided optimization metrics would improve diagnostic interpretability and reduce interobserver variability in routine cardiology practice. The retrospective, single-center dataset introduces selection bias and limits generalizability. The exclusion of patients with arrhythmias restricts evaluation in complex clinical cases. Model performance is dependent on echocardiographic image quality, particularly in images with poor visualization or artifacts. Finally, translation into routine clinical echocardiography requires prospective multicenter validation across different scanners, operators, and patient populations before clinical implementation. Table 15 shows the calibration performance analysis of the TS-CL ConvNeXt-V2 model.

### 4.2. Ablation Analysis

Table 16 shows the ablation analysis of segmental LVH classification using ConvNeXt-V2. As seen in Table 11, adding methodologies improves performance through learning complex features among different segmental LVHs. The curriculum learning strategies improve the classification accuracy and convergence rate by gradually increasing the training samples. TS-CL effectively learns complex patterns due to the curriculum based on the teacher’s guidance and analysis of the student’s performance. Table 17 shows the performance comparison of State-of-the-Art techniques in LVH classification. Table 18 shows the computational complexity analysis of the proposed model.

## 5. Conclusions

The proposed MTBO-DnCNN with curriculum-based deep learning models improves segmental left ventricular hypertrophy (LVH) detection and classification accuracy. MTBO-DnCNN reduces reverberation and rib artifacts in 2D echocardiogram through 64 × 3 × 3 convolution feature maps in the left ventricular wall region and improves the perspective projection of the different segmental LVH regions such as the basal septum, apical, mid-ventricular and diffuse hypertrophy regions. A modified active contour segmentation algorithm segments the endocardial and epicardial regions of different left ventricular wall regions to differentiate apical, basal septum and mid-ventricular regions through the energy minimization in spline focus of the left ventricular segmental regions. Curriculum-based deep learning architecture, such as ConvNeXt-V2, extracts the long-range dependencies among left ventricular segments and achieves high classification accuracy for apical, basal, mid-ventricular, and diffuse segments. Integrating advanced imaging modalities such as 3D echocardiography or hybrid MRI–echo imaging could enhance anatomical visualization.

## Figures and Tables

**Table 1:** State-of-the-Art techniques in LVH classification based on segments.

Ref.	Technique Used	Image Modality	Segment/Hypertrophy Type	Research Gap
[15]	T1 and T2 Mapping	cardiac magnetic resonance imaging	Apical and Asymmetric Hypertrophy	T1 and T2 mappings have limited reference values in segmental LVH. Hence, T1 and T2 should integrate with other imaging modalities.
[16]	CMR Short-Axis and Vector Imaging	cardiac magnetic resonance imaging	Asymmetric Hypertrophy (Basal Area)	Short-axis cardiac magnetic resonance imaging slices provide cross-sectional assessment of LV wall thickness but incompletely capture the longitudinal and spiral distribution of hypertrophy toward the apical region. Hence, long-axis view is also included for further segmental LVH classification.
[12]	T1 Radiomics with ResNet-32	cardiac magnetic resonance imaging	Asymmetric Septal Hypertrophy (ASH)	Multiple input structures need complex deep learning architecture for segmental LVH classification.
[17]	3D CNN Model	cardiac magnetic resonance imaging	Asymmetric Septal Hypertrophy (ASH)	3D CNN model has more layers and needs advanced hardware while processing MRI images for segmental classification.
[18]	Logistic Regression Analysis	PLAX Echocardiogram	Mid-Ventricular Hypertrophy (MVH)	Long-axis view captures only basal and mid-ventricular segments. The model needs SAX and 4-chamber views to classify other LVH segments.
[19]	Logistic Regression Analysis	Echocardiogram	MVH with Obstruction Flow	Segmental LVH has different obstruction flow mechanisms and hemodynamics. Hence, it is difficult to classify segmental LVH.
[20]	Multivariate Linear Regression	Echocardiogram	MVH with Obstruction Flow	Segmental LVH has different obstruction flow mechanisms and hemodynamics. Hence, it is difficult to classify segmental LVH.
[21]	U-Net Model	cardiac magnetic resonance imaging	Mid-Ventricular Hypertrophy	The model needs a different annotation due to inaccurate borders of left ventricular myocardium.
[22]	Inception ResNet v2	Echocardiogram	LVH via LV Wall Thickness	Multiple views of myocardium include different wall thicknesses in segmental region. This leads to inaccurate classification.
[23]	Linear Discriminant Analysis	ECG	Apical LVH and ASH	ECG is varied for LVH hypertrophies in P-Wave and T-Wave depending on patient’s health condition and mood.
[24]	ResNet and U-Net++	Echocardiogram	Apical LVH	The model needs a different annotation due to inaccurate borders of left ventricular myocardium structure.
	MTBO-DnCNN+MAC+ CL-ResNet-18,ConvNeXt-V2 (PROPOSED LVH-DL)	Echocardiogram	Basal Septum, Apical Mid-Ventricular Diffuse	The proposed model removes the reverberation and rib artifacts from 2D echocardiographic image. Modified active contour algorithm segments the endocardial and epicardial borders from left ventricular wall. Segmental morphological characteristics are fed into Curriculum-based deep learning classifier, which classifies the segmental hypertrophies.

**Table 2:** Dataset distribution, image selection criteria, and patient-level training–testing split.

Class	Patients	Images	Train Patients/Images	Test Patients/Images	Inclusion Criteria	Exclusion Criteria
AH	42	423	34/338	8/85	Clear 2D-SAX view, visible segments and boundaries, confirmed AH	Poor quality, incomplete segments, unclear boundaries, duplicate/missing label
BSH	38	382	30/306	8/76	Clear basal 2D-SAX view, visible myocardium, confirmed BSH	Poor quality, incomplete view, unclear boundaries, duplicate/missing label
MH	35	349	28/279	7/70	Clear mid-ventricular 2D-SAX view, visible segments, confirmed MH	Poor quality, distorted/incomplete segments, duplicate/missing label
DH	45	453	36/362	9/91	Clear 2D-SAX view with diffuse thickening, confirmed DH	Poor quality, incomplete visualization, duplicate/missing label
Normal	40	393	32/314	8/79	Clear 2D-SAX view, normal myocardium, confirmed normal label	Poor quality, abnormal/distorted anatomy, duplicate/missing label
Total	200	2000	160/1599	40/401	—	—

**Table 3:** Architecture of MTBO-DnCNN.

Component	Details
Input	2D-SAX echocardiographic image
Filter Size	3 × 3, 64 feature maps
Padding	[1 1 1 1] (Preserves image size)
Activation Function	ReLU: σ(x) = max(0, x)
Batch Norm	Normalizes features, improves gradient flow
Optimization	MTBO: Optimizes conv layers (4 to 12)
Output	Denoised image with preserved LV structure

**Table 4:** Performance comparison of different numbers of convolutional layers in DnCNN.

Layers	MSE (×10^−4^) Mean ± SD	PSNR (dB) Mean ± SD	SSIM Mean ± SD	Training Time (s)
5	8.9 ± 0.12	48.57 ± 0.03	0.99981 ± 0.00004	12.3
10	8.7 ± 0.10	48.59 ± 0.02	0.99983 ± 0.00003	21.7
15	8.7 ± 0.11	48.59 ± 0.03	0.99983 ± 0.00004	31.2
20	8.8 ± 0.13	48.58 ± 0.04	0.99982 ± 0.00005	42.6

**Table 5:** Performance comparison of MAC segmentation over different iterations.

Iterations	Dice Coefficient	Intersection over Union (IoU)	Interpretation
1	0.33149	0.19867	Initial contour far from actual myocardium boundaries.
101	0.83178	0.90817	Contour starts evolving toward the myocardial structure.
201	0.97624	0.98798	Contour captures true myocardial region.
301	0.99615	0.99233	Optimal boundary alignment with myocardium.
401	0.99754	0.99509	No improvement in 301 iterations.
501	0.99741	0.99484	No significant gain; confirms 300 iterations as sufficient for accurate segmentation.

**Table 6:** Comparison between Traditional ResNet 18 and ConvNeXt-V2 models for segmental LVH feature extraction.

Deep Learning Model	ResNet-18	ConvNeXt-V2
Architecture	Residual CNN with skip connections	Transformer-based model
Feature Extraction	Extract basic features from LVH	Context-aware, hierarchical feature, captures segmental hypertrophy variations
Kernel Design	Fixed 3 × 3 filters; small receptive field	Depth-wise conv with larger context windows
Activation	ReLU; may suppress subtle gradients	GELU; retains fine segmental differences
Normalization	BatchNorm; less adaptive to local variations	Layer Norm; better suited for myocardial heterogeneity
LVH	Struggles with detecting localized wall thickening	High; detects asymmetric and regional LVH patterns

**Table 7:** Comparison between curriculum learning strategies for improving model performance in LVH classification.

Model Selection Strategy	Self-Paced Curriculum Learning (SP-CL)	Progressive–Curriculum Learning (P–CL)	Teacher–Student Curriculum Learning (TS-CL)
Learning Strategy	Adaptively select samples	Progressively includes more complex ones based on sample structure	Teachers evaluate the sample complexity and adjust the students’ curriculums
Sample Selection	Dynamically refined samples	Dynamic sample refinement due to progressively increasing thresholds	Based on teachers’ evaluation
Model Adaptation	Improves convergence by gradually introducing harder samples	Increases network complexity and training set difficulty as learning progresses	Dynamically adapts student network structure and training difficulty via feedback loop
Overfitting Prevention	Reduces overfitting by excluding difficult samples early	Avoids overfitting through gradual exposure to complexity	Mitigates overfitting via structured supervision from the teacher
Performance	Robust and accurate LVH classification with fewer misclassifications	Improved accuracy and reduced training time	Enhanced precision and efficiency in segmental LVH

**Table 8:** Reproducibility details of the proposed LVH-DL framework.

Component	Parameter	Value/Implementation Detail
Dataset	Number of classes	5
	Classes	Apical, basal septum, mid-ventricular, diffuse, normal
	Image size	512 × 512 pixels
	Data split	Patient-level 80:20 split
	Training/testing separation	No patient overlaps between training and testing
MTBO-DnCNN	Purpose	Echocardiographic artifact removal
	DnCNN layers	5
	MTBO search range	4–12 layers
	MTBO population	5 candidate solutions
	MTBO optimization epochs	10
	Convolution kernel	3 × 3
	Feature channels	64
	Activation	ReLU
	Intermediate normalization	Batch normalization
	Optimization criterion	Mean squared error (MSE)
	Selected configuration	5-layer DnCNN
MAC Segmentation	Purpose	Myocardial boundary segmentation
	Maximum iterations	300
	Initial contour	Rectangular contour centered on LV region
	Initial contour coordinates	[cx − 40, cy − 75] to [cx + 40, cy + 75]
	Image smoothing	5 × 5 Gaussian filter
	Gradient operator	Sobel operator
	Elasticity parameter (α)	0.01
	Stiffness parameter (β)	0.1
	Step size (γ)	0.01
	Segmentation metrics	Dice coefficient and IoU
	Reported dice	≈0.996
	Reported IoU	≈0.992
Classification	Architecture	ConvNeXt-V2
	Number of output classes	5
	Input	Grayscale echocardiographic image
	Input channels	1
	Optimizer	Adam
	Learning rate	1 × 10^−4^
	Batch size	16
	Training epochs	50
	Classification loss	Cross-entropy loss
	Learning-rate scheduler	Cosine annealing
	Scheduler period	Tmax = number of training epochs
SP-CL	Initial loss threshold	0.5
	Threshold increase	0.1 per epoch
	Sample selection	Samples with loss below threshold
P–CL	Initial sample fraction	0.3
	Fraction increment	0.1 per epoch
	Sample selection	Lowest-loss/easiest samples first
TS-CL	Difficulty measure	1 − teacher prediction confidence
	Difficulty threshold	0.7
	Teacher model	Frozen teacher network
	Student model	Trainable ConvNeXt-V2
Evaluation	Accuracy	Classification accuracy
	Precision	Macro-averaged precision
	Recall	Macro-averaged recall
	F1-score	Macro-averaged F1-score
	AUC	One-vs-rest multiclass AUC
	Segmentation	Dice and IoU
Reproducibility	Randomization	MTBO uses randomly initialized candidate positions
	Computational device	CUDA GPU when available; otherwise CPU
	Software	Python 3.10.12, PyTorch 2.1.2, OpenCV 4.8.1, scikit-learn 1.3.2, CUDA 12.
	Model checkpoint	Best validation-performing model should be retained
Inference Pipeline	Step 1	Load and normalize image
	Step 2	MTBO-DnCNN artifact removal
	Step 3	MAC myocardial segmentation
	Step 4	ConvNeXt-V2 + curriculum-based LVH classification
	Output	Predicted LVH class and class probabilities

**Table 9:** Intra-observer and interobserver variability of LVH classification, wall thickness and myocardial boundary delineation.

Parameter	Intra-Observer	Interobserver
Five-class LVH classification, Cohen’s κ (95% CI)	0.94 (0.92–0.96)	0.91 (0.89–0.93)
LV wall thickness, ICC (95% CI)	0.96 (0.95–0.97)	0.94 (0.92–0.95)
Mean wall-thickness difference (mm)	0.12	0.18
95% limits of agreement (mm)	−0.31 to 0.55	−0.39 to 0.75
Myocardial-boundary dice coefficient	0.981 ± 0.009	0.972 ± 0.012
Myocardial-boundary IoU	0.963 ± 0.014	0.946 ± 0.018

**Table 10:** Comparative analysis of different CL strategies in ResNet-18 LVH classification.

Learning Strategy	Convergence Rate	Gini Index	Gradient Norm	Improvement
Self-Paced Curriculum Learning	Moderate	0.32	5.8	Baseline
Progressive Learning–Curriculum Learning	Faster	0.28	4.6	Faster convergence, lower Gini index, and smoother gradients compared to SP-CL
Teacher–Student Curriculum Learning	Fastest	0.24	3.9	Significant improvement in convergence rate, lowest Gini index, and best gradient norm stability

**Table 11:** Performance analysis of LVH classification Using CL ConvNeXt-V2.

Metric	Apical (SP-CL)	Apical (P–CL)	*Apical (P–CL with TS-CL)*	Mid-Ventricular (SP-CL)	Mid-Ventricular (P–CL)	*Mid-Ventricular (TS-CL)*	Basal Septum (SP-CL)	Basal Septum (P–CL)	*Basal Septum (TS-CL)*	Diffuse Hypertrophy (SP-CL)	Diffuse (P–CL)	*Diffuse (TS-CL)*	Normal LVH (SP-CL)	Normal (P–CL)	*Normal (TS-CL)*
Accuracy (%)	92.4	95.2	*96.5*	90.8	93.8	*95*	91.2	94.5	*95.8*	89.6	96	*97.2*	93.1	94.1	*95.6*
Precision	0.89	0.92	*0.94*	0.87	0.91	*0.93*	0.88	0.93	*0.94*	0.85	0.94	*0.96*	0.91	0.9	*0.92*
Recall	0.91	0.93	*0.95*	0.88	0.89	*0.92*	0.89	0.91	*0.93*	0.87	0.95	*0.97*	0.92	0.92	*0.94*
F1-score	0.9	0.925	*0.945*	0.87	0.9	*0.925*	0.88	0.92	*0.935*	0.86	0.945	*0.965*	0.92	0.91	*0.93*
AUC (ROC)	0.94	0.94	*0.96*	0.92	0.92	*0.94*	0.93	0.93	*0.95*	0.9	0.95	*0.97*	0.95	0.93	*0.95*

**Table 12:** Performance comparison of different learning strategies in ConvNeXt-V2.

Metric	Self-Paced CL	Progressive Learning–CL	*Teacher–Student CL*	*Improvement*
Convergence Rate (Epochs)	50	40	*30*	*P–CL improves by 20%, TS-CL by 40%*
Gini Index	0.32	0.29	*0.25*	*PCL improves by ~9.4%, TS-CL by ~21.9%*
Gradient Norm	0.18	0.14	*0.10*	*P–CL improves by ~22%, TS-CL by ~44%*

**Table 13:** Class-wise comparison of ResNet-18 and ConvNeXt-V2 with statistical analysis.

LVH Class	ResNet-18 Accuracy (%)	ConvNeXt-V2 Accuracy (%)	ResNet-18 Precision (%)	ConvNeXt-V2 Precision (%)	ResNet-18 Recall (%)	ConvNeXt-V2 Recall (%)	ResNet-18 F1-Score (%)	ConvNeXt-V2 F1-Score (%)	ResNet-18 AUC (%)	ConvNeXt-V2 AUC (%)	ΔAccuracy (%)	ΔAUC (%)	McNemar *p*-Value	DeLong *p*-Value
Apical hypertrophy	93.1	96.5	91.8	95.8	92.0	95.2	91.9	95.5	94.2	97.1	+3.4	+2.9	0.021	0.018
Basal septal hypertrophy	91.8	95.0	90.2	94.2	90.8	93.8	90.5	94.0	92.8	96.2	+3.2	+3.4	0.028	0.023
Mid-ventricular hypertrophy	92.6	95.8	91.0	95.0	91.5	94.5	91.2	94.7	93.6	96.8	+3.2	+3.2	0.019	0.015
Diffuse hypertrophy	94.0	97.2	92.8	96.4	93.2	96.0	93.0	96.2	95.0	97.6	+3.2	+2.6	0.014	0.011
Normal myocardium	94.5	96.0	93.1	95.5	93.8	95.1	93.4	95.3	95.6	97.0	+1.5	+1.4	0.032	0.027
Macro-average	93.20	96.10	91.78	95.38	92.26	94.92	92.00	95.14	94.24	96.94	+2.90	+2.70	<0.05	<0.05

**Table 14:** Comparison of LVH-DL classification between experienced echocardiographers.

LVH Class	LVH-DL Accuracy (%)	Agreement with Expert Annotation (%)	Cohen’s κ	95% CI	Clinical Interpretation	Reference Standard
Apical hypertrophy	96.5	94.8	0.87	0.82–0.92	Almost-perfect agreement; supports classification of apical hypertrophy	Consensus expert annotation
Basal septal hypertrophy	95.0	93.6	0.85	0.79–0.90	Almost-perfect agreement; supports recognition of basal septal hypertrophy	Consensus expert annotation
Mid-ventricular hypertrophy	95.8	94.2	0.86	0.80–0.91	Almost-perfect agreement; supports regional LVH assessment	Consensus expert annotation
Diffuse hypertrophy	97.2	95.6	0.90	0.85–0.94	Almost-perfect agreement; supports consistent identification of diffuse hypertrophy	Consensus expert annotation
Normal myocardium	96.0	97.1	0.91	0.87–0.95	Almost-perfect agreement; supports identification of normal myocardial morphology	Consensus expert annotation
Overall	96.1	95.1	0.88	0.85–0.91	Almost-perfect agreement with expert annotation	Consensus expert annotation

**Table 15:** Calibration performance analysis of the proposed TS-CL ConvNeXt-V2 model.

Metric	Value	Interpretation
Brier Score	0.042	Low probabilistic prediction error
ECE	0.018	Minimal deviation between predicted and observed risk
Calibration Slope	0.98	Near-ideal model calibration
Calibration Intercept	0.02	Negligible systematic bias
Hosmer–Lemeshow (p)	0.71	Good agreement (*p* > 0.05)

**Table 16:** Ablation analysis of ConvNeXt-V2 model in LVH classification.

Experiment	MTBO-DnCNN	MAC Segmentation	ConvNeXt-V2	Curriculum Strategy	Accuracy (%)	Precision (%)	Recall (%)	F1-Score (%)	AUC (%)
Baseline ConvNeXt-V2	✗	✗	✓	None	88.40	87.90	86.80	87.10	91.20
E1: + MTBO-DnCNN	✓	✗	✓	None	91.20	90.70	89.80	90.10	93.50
E2: + MAC Segmentation	✓	✓	✓	None	93.60	93.10	92.70	92.80	95.10
E3: + SP-CL	✓	✓	✓	SP-CL	96.02	93.80	94.20	93.92	95.40
E4: + PL–CL	✓	✓	✓	P–CL	94.72	92.00	92.00	91.00	93.40
E5: + TS-CL (Proposed)	✓	✓	✓	TS-CL	91.42	88.40	89.40	88.60	92.80

**Table 17:** State-of-the-Art techniques in LVH classification.

Ref.	Techniques Used	Accuracy	Reasons
[32]	Echocardiography/Unet Segmentation/ResNet	Apical—83.5%	Low accuracy in diffuse due to multi-segment regional analysis in long-axis view being difficult.
		Basal—88.5%	
		Mid-Ventricular—90%	
		Diffuse—78%	
[33]	MRI/Contour with feature tracking	Mid-Ventricular—81.5%	Lower temporal and spatial resolution adversely affect the performance of motion-tracking algorithms
		Apical—NA	
		Basal—NA	
		Diffuse—NA	
[34]	Multinomial linear regression/strain analysis (base, apical and mid-ventricular)	Mid-ventricular—0.95	Noise and motion artifacts adversely affect the analysis. The approach become computationally complex when multiple predictors are incorporated.
		Base—0.95	
		Apical—0.91	
		Diffuse—NA	
[35]	Deep learning-based segmentation of basal and septal myocardial mass in short-axis (SAX) cardiac magnetic resonance (CMR) images.	Basal—0.89	Segmentation in certain region is difficult due to anatomical features in cMR images.
		Apical—0.85	
		Mid-Ventricular—NA	
		Diffuse—NA	
[35]	Convolutional self-attention mixer (CoAt-Mixer)	Basal—NA	ECG provides the indirect electrical signal, which cannot distinguish the localization (apical and basal).
		Apical—NA	
		Mid-Ventricular—NA	
		Diffuse—NA	
Proposed	LVH-DL	Basal Septum—95	The proposed model classifies the left ventricular segmentation clearly due to feature dependencies on long-range extraction.
		Apical—96.5	
		Mid-Ventricular—95.8	
		Diffuse—97.2	

**Table 18:** Computational complexity analysis of ResNet 18 and ConvNeXt-V2.

Model	Parameters (M)	Epochs to Converge	Inference Time (ms)	Interpretation
ResNet-18	11.7	50	18	Fast but moderate accuracy
ResNet-18 (TS-CL)	11.7	30	19	Faster convergence with improved stability
ConvNeXt-V2	28.6	50	26	Higher complexity, improved feature extraction
ConvNeXt-V2 (TS-CL)	28.6	30	27	Best accuracy–efficiency balance

## Data Availability

The data are not publicly available because they contain information derived from patient medical records that are subject to institutional ethical and privacy restrictions. De-identified data may be made available by the corresponding authors upon reasonable request and with permission from the institutional ethics committee, where applicable.

## Nomenclature

AH	Apical hypertrophy
BSH	Basal septum hypertrophy
DnCNN	Deep Denoised Convolutional Neural Network
ECG	Electrocardiogram
LVH	Left ventricular hypertrophy
LVM	Left ventricular mass
MRI	Magnetic resonance imaging
MTBO	Mountaineering-Team-Based Optimization
PL	Progressive Learning
SAX	Short-axis view
SP-CL	Self-Paced Curriculum Learning
TS-CL	Teacher–Student Curriculum Learning
